# Dietary Supplementation With Eicosapentaenoic Acid Inhibits Plasma Cell Differentiation and Attenuates Lupus Autoimmunity

**DOI:** 10.3389/fimmu.2021.650856

**Published:** 2021-06-15

**Authors:** Azusa Kobayashi, Ayaka Ito, Ibuki Shirakawa, Atsushi Tamura, Susumu Tomono, Hideo Shindou, Per Niklas Hedde, Miyako Tanaka, Naotake Tsuboi, Takuji Ishimoto, Sachiko Akashi-Takamura, Shoichi Maruyama, Takayoshi Suganami

**Affiliations:** ^1^ Department of Molecular Medicine and Metabolism, Research Institute of Environmental Medicine, Nagoya University, Nagoya, Japan; ^2^ Department of Nephrology, Nagoya University Graduate School of Medicine, Nagoya, Japan; ^3^ Department of Immunometabolism, Nagoya University Graduate School of Medicine, Nagoya, Japan; ^4^ Department of Organic Biomaterials, Institute of Biomaterials and Bioengineering, Tokyo Medical and Dental University (TMDU), Tokyo, Japan; ^5^ Department of Microbiology and Immunology, Aichi Medical University School of Medicine, Nagakute, Japan; ^6^ Department of Lipid Signaling, National Center for Global Health and Medicine, Tokyo, Japan; ^7^ Department of Medical Lipid Science, Graduate School of Medicine, The University of Tokyo, Tokyo, Japan; ^8^ Laboratory for Fluorescence Dynamics, Beckman Laser Institute and Medical Clinic, Department of Pharmaceutical Sciences, University of California Irvine, Irvine, CA, United States; ^9^ Department of Nephrology, Fujita Health University Graduate School of Medicine, Toyoake, Japan

**Keywords:** fatty acid, membrane dynamics, systemic lupus erythematosus, plasma cells, autoantibody, immunometabolism, autoimmunity

## Abstract

Accumulating evidence suggests that cholesterol accumulation in leukocytes is causally associated with the development of autoimmune diseases. However, the mechanism by which fatty acid composition influences autoimmune responses remains unclear. To determine whether the fatty acid composition of diet modulates leukocyte function and the development of systemic lupus erythematosus, we examined the effect of eicosapentaenoic acid (EPA) on the pathology of lupus in drug-induced and spontaneous mouse models. We found that dietary EPA supplementation ameliorated representative lupus manifestations, including autoantibody production and immunocomplex deposition in the kidneys. A combination of lipidomic and membrane dynamics analyses revealed that EPA remodels the lipid composition and fluidity of B cell membranes, thereby preventing B cell differentiation into autoantibody-producing plasma cells. These results highlight a previously unrecognized mechanism by which fatty acid composition affects B cell differentiation into autoantibody-producing plasma cells during autoimmunity, and imply that EPA supplementation may be beneficial for therapy of lupus.

## Introduction

Systemic lupus erythematosus (SLE) is a chronic systemic autoimmune disease that affects multiple organs, and it is characterized by autoantibody production. As the concordance rate for SLE in identical twins is only 25-60%, this complex disease is caused by both genetic and environmental factors (1). Patients with SLE have increased risk of atherosclerosis, and cardiovascular disease is one of the major causes of morbidity and mortality in these patients (2). It has also been reported that 36% of patients with newly diagnosed SLE have hypercholesterolemia (3), suggesting a relationship between the dysregulation of lipid metabolism and autoimmune responses. Consistently, recent genome-wide association studies and expression quantitative trait loci analyses have revealed that genes involved in lipid metabolism increase the susceptibility to autoimmune diseases such as rheumatoid arthritis and SLE (4–6). However, the molecular mechanism by which lipid metabolism influences the pathology of SLE is unclear.

Polyunsaturated fatty acids are essential nutrients that affect chronic inflammatory diseases such as metabolic syndrome and cancer by regulating lipid metabolism and their immunomodulatory effects (7). Among others, the role of omega-3 polyunsaturated fatty acid eicosapentaenoic acid (EPA), which is enriched in fish oil, in innate immune responses, including its anti-inflammatory and pro-resolving effects, have been extensively studied (8, 9). On the contrary, the effect of EPA on adaptive immune responses is poorly studied. Although a number of clinical studies have been conducted to determine whether EPA can be used to prevent or treat SLE, the results are not conclusive (10). In a few animal models of systemic lupus, dietary EPA supplementation provided beneficial effects on survival and disease severity (10, 11), however, its mechanism of action remains undetermined. In particular, although autoantibody production is a central pathogenesis of SLE, the effects of EPA on B lymphocyte function are largely unknown.

Several lines of evidence in mice deficient in cholesterol metabolism indicate that cholesterol accumulation in immune cells promotes lymphocyte proliferation and lupus autoimmunity (12–15). Mice lacking liver X receptors (LXRα and LXRβ), which are pivotal regulators of lipid homeostasis, develop age-dependent lupus-like autoimmunity, and treatment with an LXR agonist ameliorated disease progression in a spontaneous lupus mouse model (16, 17). Recently, we demonstrated that cholesterol overload in CD11c^+^ antigen-presenting cells (APCs) causes systemic autoimmunity in LXR-deficient mice by stimulating the production of the B cell growth factors, B cell activating factor (BAFF) and April, which support B cell expansion and autoantibody production (14). As an underlying mechanism, it is likely that increased cholesterol content in cellular membranes enhances the lipid raft-dependent signaling of immune pathways such as toll-like receptor (TLR) signaling. In support of this, pharmacological activation of LXRs reduces cellular cholesterol content by inducing ATP-binding cassette protein A1 (ABCA1), which creates a dynamic membrane environment and blunts inflammatory signaling (18). In addition to cholesterol metabolism, LXRs also regulates fatty acid and phospholipid metabolism (17). For instance, LXR-induced lysophosphatidylcholine (lysoPC) acyltransferase 3 increases the abundance of polyunsaturated fatty acids in phospholipids and membrane fluidity (19). These findings led us to hypothesize that changing the cellular fatty acid composition in immune cells modulates membrane dynamics and influences the inflammatory signaling and pathology of lupus.

In this study, we demonstrated that dietary EPA supplementation in two lupus mouse models, namely imiquimod (IMQ)-induced and spontaneous C57BL/6*^lpr/lpr^* models, attenuates autoantibody production and immunocomplex deposition in kidney glomeruli. In addition to the anti-inflammatory effect of EPA in APCs, we discovered that EPA suppresses the differentiation of naïve B cells into autoantibody-producing plasma cells. Dietary EPA supplementation increased its abundance in B cell membrane, thereby increasing their fluidity and attenuating the signal for plasma cell differentiation. These results highlight a mechanism by which cellular fatty acids regulate the function of B lymphocytes in the context of systemic autoimmunity.

## Materials and Methods

### Resources and Primers for Q-PCR

Information of key resources and primers for Q-PCR used in this study are shown in **Tables S1**, **S2**.

### Animal Studies

Female C57BL/6J*^+/+^* mice and C57BL/6J*^lpr/lpr^* mice were purchased from Japan SLC, and female FVB/N mice were purchased from CLEA Japan. Eicosapentaenoic acid (EPA) ethyl ester was provided by Mochida Pharmaceutical. All animals were housed in a temperature-controlled animal room under 12 h light/12 h dark cycle under pathogen-free conditions. To induce lupus, FVB/N mice at 6 to 10 week of age were received a dayily topical dose of 25 mg of Beselna cream (5% imiquimod, Mochida Pharmaceutical) or control vaseline on the right ear 3 times a week. The mice were fed fish meal-free diet supplemented with 5% palmitic acid or EPA for 6 weeks from 4 week-old. All of the animals were handled according to approved guide for the care and use of laboratory animals (ILAR Guideline). All animal experiments were approved by the Committee on the Ethics of Animal Experiments of Nagoya University (No. 20253).

### Flow Cytometry

Spleens were digested with 1 mg/ml Collagenase type IV (Worthington) and 40 U/ml DNase I (Roche Diagnostics) in HBSS (−) at 37°C for 30 min. Single-cell suspension were incubated with anti-mouse CD16/32 antibody (BioLegend) to prevent nonspecific binding of antibodies and stained with fluorescence-conjugated antibodies in 0.5% BSA, 5 mM EDTA in PBS. The following conjugated antibodies were used for the staining: B220 (RA3-6B2), CD23 (B3B4), CD21 (7E9), CD138 (281-2), CD95 (SA367H8), GL7 (GL7), CD3 (145-2C11), CD4 (GK1.5), CD8 (53-6.7), CD11c (N418), MHCII (M5/144.15.2), PDCA (9.27E+02), CD11b (M1/70), Ly6G (1A8), CD44 (IM7), CD62L (MEL-14), PD-1 (29F.1A12), CD25 (3C7) (BioLegend), Peanut Agglutinin (PNA, Vector Laboratories), and DAPI (BioLegend). For intracellular staining, cells were incubated with Transcription Factor Fix/Perm Working Buffer (TONBO Biosciences) at room temperature for 30 min. After washing twice with Flow Cytometry Perm Buffer (TONBO Biosciences), cells were stained with APC-conjugated anti-Blimp1 (5E7, BioLegend) or PE-conjugated anti-Foxp3 (3G3, TONBO Biosciences) at room temperature for 20 min in the dark. After washing, cells were analyzed on MACSQuant Analyzer (Miltenyi Biotec) and FlowJo software (BD Bioscience). An example of the gating strategy for B cells, T cells and myeloid cells is shown in **Figures S2**, **S5**.

### Histology

Kidneys were collected and embedded in OCT compound and frozen on dry ice. Three μm frozen-sections were air-dried, fixed in acetone, blocked with 6% BSA and 4% normal goat serum in PBS, and stained with FITC-conjugated AffiniPure goat anti-mouse IgG (H+L) (Jackson ImmunoResearch) and FITC-conjugated goat IgG fraction to mouse complement C3 (MP Biomedicals). Sections were mounted using ProLong Gold Antifade Reagent with DAPI (Invitrogen). Evaluation of the fluorescence intensity of IgG and C3 was performed by scoring the intensity of staining for individual glomeruli as 0 (negative), 1 (positive above background), 2 (positive), and 3 (brightly positive) for at least 10 glomeruli per section. Spleens were collected and fixed with neutral buffered−formalin and embedded in paraffin. Five μm–thick sections were stained with hematoxylin and eosin. Images were obtained using BZ-X700 fluorescence microscope (KEYENCE).

### Cytokine Measurement

Serum levels of total IgG, total IgM (Invitrogen), autoantibodies against nuclear, histone (Alpha Diagnostic International) and dsDNA (FUJIFILM Wako Shibayagi), and BAFF (R&D Systems), were measured by ELISA kits. Cell-free culture medium was collected and the levels of IL12p70 (Invitrogen), IL6 and TNFα (R&D Systems) were measured by ELISA kits. IFNα/β amount in serum and culture medium was quantified using IFN sensor-B16-Blue IFNα/β cells (bb-ifnt1, InvivoGen) according to manufacturer’s instruction. Briefly, mouse serum diluted 1:10 or cell-free culture medium was combined with RPMI 1640 supplemented 10% FBS containing 2 × 10^4^ cells in each well of a 96-well plate and incubated at 37°C with 5% CO_2_ for 20 to 24 h. B16-Blue IFNα/β cell supernatant was incubated with QUANTI-Blue (InvivoGen) and secreted embryonic alkaline phosphatase (SEAP) levels were determined using a spectrophotometer at 620 nm.

### Cell Culture

Murine bone marrow-derived dendritic cells (BMDCs) were obtained as described previously (14). Bone marrow cells were obtained from femurs and tibias and 2 × 10^6^ cells were cultured in RPMI 1640 supplemented with 10% FBS, 15% GM-CSF conditioned medium, 100 U/ml penicillin, 100 mg/ml streptomycin, 2 mM L-glutamine, 1 mM Sodium pyruvate, 50 μM 2-ME in 10 cm petri dish for 7 days. BMDCs (1 × 10^6^ cells) were seeded per well in RPMI containing 10% FBS, 100 U/ml penicillin and 100 mg/ml streptomycin in 12-well plate and treated with ethanol, 50 μM palmitate (Sigma, P5585) or 50 μM EPA (Sigma, E6627) with 5 μM BSA overnight. Cells were then stimulated with 10 ng/ml LPS (Sigma) for 4 or 24 h or 5 ng/ml IFNγ (PeproTech) for 6 h. APCs, naïve B cells and pan-B cells were isolated using CD11c MicroBeads UltraPure, B Cell Isolation Kit and Pan B Cell Isolation Kit II (Miltenyi Biotec) respectively, according to the manufacturer’s instructions. For B cell differentiation assay, naïve B cells (5 × 10^4^ cells) from spleen were seeded per well in RPMI 1640 containing 10% FBS, 100 U/mL penicillin, 100 mg/ml streptomycin and 50 μM 2-ME in 96-well plate and treated with 1 μg/ml anti-CD40 antibody (HM40-3, BioLegend) and 3 ng/ml recombinant murine IL4 (PeproTech) in the presence of ethanol, palmitate or EPA with 5 μM BSA for 96 h. Naïve B cells were also co-cultured with CD11c^+^ cells from spleen and stimulated with 1 μg/ml anti-CD40 antibody and 1 μg/ml R848 (AdipoGen) in the presence of ethanol or 50 μM EPA with 5 μM BSA for 96 h.

### Gene Expression

Total RNA was isolated from cells and tissues with Sepazol (Nacalai Tesque) according to the manufacturer’s instruction. Five hundred ng of total RNA was used for cDNA synthesis, and gene expression was quantified by real-time PCR using SYBR Green and StepOne Plus Instrument (Applied Biosystems). Gene expression levels were normalized to *36B4*. Primer sequences are listed in **Table S3**.

### Lipid Analysis by Gas Chromatography/Mass Spectrometry (GC/MS)

Cells were snap frozen in liquid nitrogen and subsequently subjected to Folch lipid extraction (20). Nonadecanoic acid (1 μg for each sample, C19:0, Matreya) was used as internal control. The organic phases were evaporated to give the residues and dissolved in dehydrated hexane (100 μl). To transesterified phospholipids and triglycerides, sodium methoxide (0.5 M in dehydrated methanol) was added to the sample, and the solutions were incubated for 30 min at 45°C. Then, 1 M hydrochloric acid, distilled water and hexane were added to the sample solutions and mixed. The organic phase was evaporated to give the residues, dissolved in the mixture of dehydrated methanol and toluene. The extracted fatty acids were methylated with trimethylsilyldiazomethane (10% in hexane) at 50°C for 1 h. After evaporation, the residues were dissolved in dehydrated hexane and subjected to GC/MS analysis. GC/MS analyses were performed on a GCMS-QP2020 (Shimadzu) using BPX-70 column (0.25 μm phase thickness, 0.22 μm internal diameter, 30 m length, SGE) and helium as a carrier gas. The oven temperature was initially held at 50°C for 1 min, increased to 150°C at a rate of 15°C/min, further increased to 230°C at a rate of 4°C/min, and finally held at 230°C for 2 min. The measurements were performed in the selected-ion monitoring (SIM) mode. The ions used for the quantification were as follows: C16:0 (14.51 min) *m/z* = 74, C18:1 (17.74 min) *m/z* = 69, C19:0 (18.59 min) *m/z* = 74, C20:4 (22.60 min) *m/z* = 79, C20:5 (23.74 min) *m/z* = 74, and C22:6 (26.97 min) *m/z* = 79.

### Lipid Analysis by Ultra High Performance Liquid Chromatography-Triple Time-of-Flight Mass Spectrometry (UHPLC-Triple-TOF/MS)

Cells were snap frozen in liquid nitrogen and subsequently subjected to lipid extraction with 200 μl of Ultrapure water (FUJI Film Wako), 200 μl of methanol (FUJI Film Wako), 400 μl of dichloromethane (FUJI Film Wako) and 20 μl internal standard solution mixture (10 μl of mouse SPLASH LIPIDOMIX mass spec Internal standard (Avanti) and 10 μl of 100 μM ^13^C16-palmitic acid was dissolved in acetonitrile). The mixture was centrifuged at 20,000 × g for 10 min, and the organic phase was collected. The remaining precipitate and aqueous phase were then mixed with 400 μl of dichloromethane, and the resulting mixture was centrifuged at 20,000 × g for 5 min to give the organic phase. The combined organic phases were then evaporated to give the residues and dissolved in a solution consisting of 100 μl of a mixture of isopropyl alcohol/acetonitrile/water (2:1:1, *v/v/v*). UHPLC-Triple TOF/MS analyses were performed on a Shimadzu UHPLC Nexera X2 system (Shimadzu) using a TSKgel ODS-120H column (1.9 μm, 100 mm × 2.0 mm, TOSOH) and a Triple TOF 5600+ (SCIEX) with an electrospray ionization device running in the positive and negative ion mode. The autosampler injection volume was set to 10 mL and the eluent flow rate to 0.4 ml/min. Mobile phase A consisted of a 0.1% (*v/v*) solution of formic acid and 10 mM ammonium formate in acetonitrile/water (60/40, *v/v*), and mobile phase B consisted of a 0.1% (*v/v*) solution of formic acid and 10 mM ammonium formate in isopropyl alcohol/acetonitrile (90/10, *v/v*). The linear gradient conditions were as follows: 40% B at 0 min, 43% B at 3 min, 55% B at 15 min, 99% B at 25 min, 99% B at 27 min and 40% B at 27.01 min, followed by a 2.99 min equilibration time. The detector conditions were as follows: ion spray voltage at 5500 V, source temperature of 350°C, ion source gas 1, 60 psi, ion source gas 2, 60 psi, declustering potential, 80 V and collision energies of 45 V, collision energy spread, 15 V. Nitrogen was used as the collision gas. The raw data “mzXML” was used to convert to “abf “ format with the ABF converter. The MS DIAL equipped with FiehnLib24 was used for raw peaks exaction, peak alignment, deconvolution analysis and identification.

### Membrane Dynamics

Membrane dynamics was analyzed as described (18, 21). Briefly, Ba/F3 cells were treated with 100 μM palmitate in RPMI1640 containing 10% FBS and 0.05% IL3 conditioned medium for 8 h. Cells were collected then 6 × 10^4^ cells were plated onto 9.5 mm multi glass-bottom dishes in RPMI1640 containing 10% FBS 0.05% IL3 conditioned medium with 100 μM palmitate or 100 μM EPA and incubated for overnight. Cells were incubated with 10 μM Laurdan (6-dodecanoyl-2-dimethylaminonaphthalene; Invitrogen) at 37°C for 30 min. Spectral data were obtained with FV1000-D IX81 confocal laser scanning microscope (Olympus) at excitation 405 nm. The emission signal was collected in two bands: 430–455 nm and 490–540 nm. Spectral data were processed by the SimFCS software (Laboratory for Fluorescence Dynamics). GP value of each pixel was used to generate the pseudocolored GP image.

### Statistical Analysis

Data were analyzed using Prism Version 9 software (GraphPad). For data with two groups, unpaired t-tests were performed as homogeneity of variance and normality were confirmed. The data were analyzed by one-way analysis of variance (ANOVA) followed by the *post hoc* Tukey-Kramer’s multiple comparison test for the comparison among 3 or more groups if homogeneity of variance and normality were confirmed, or Kruskal-Wallis test followed by *post hoc* Dunn’s multiple comparison test if not confirmed. Data are presented as the mean ± SEM. p < 0.05 was considered statistically significant.

## Results

### EPA Supplementation Attenuates Autoimmunity in Imiquimod-Induced Lupus Mice

**Figure 1:**
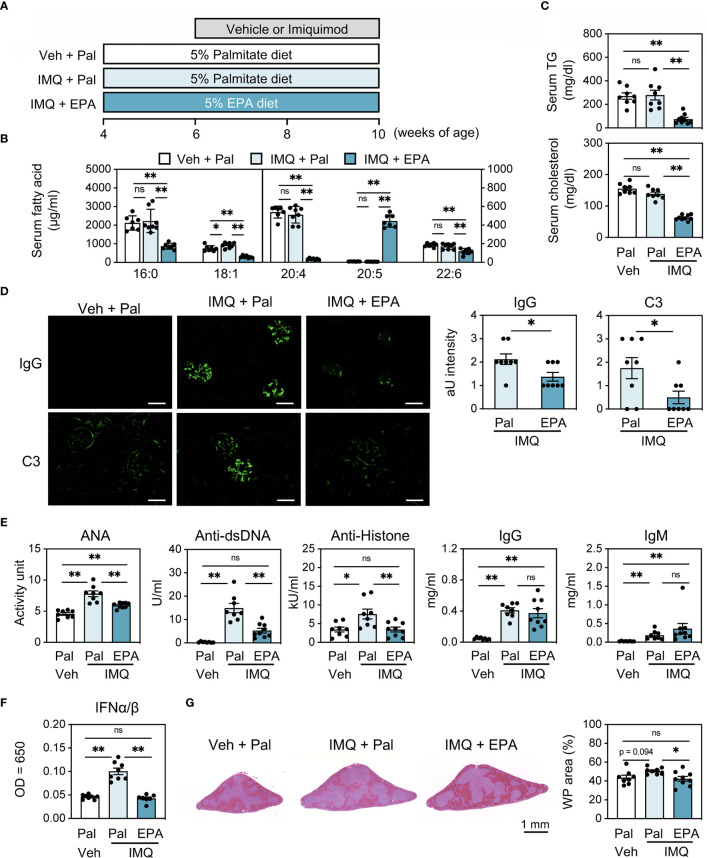
EPA supplementation attenuates autoimmunity in imiquimod (IMQ)-induced lupus mice. **(A)** Experimental protocol. Four week-old FVB/N mice were fed chow diet supplemented with 5% (*wt/wt*) palmitate (Pal) or eicosapentaenoic acid (EPA) for 6 weeks. To induce lupus, the mice received IMQ or vehicle (Veh) treatment 3 times a week during the last 4 weeks. **(B, C)** Serum levels of fatty acid **(B)**, triglyceride (TG) (**C**, top) and total cholesterol (**C**, bottom). **(D)** Immunostaining of FITC-labeled anti-mouse IgG or C3 in kidney (left) and quantification of IgG and C3 deposition (right). Original magnification: × 400, Scale bars represent 50 μm. **(E)** Serum levels of anti-nuclear antibodies (ANA), anti-dsDNA antibodies, anti-histone antibodies, total IgG and IgM. **(F)** Serum IFNα/β levels. **(G)** H&E staining of spleen sections (left) and quantification of white pulp (WP) area in whole spleen area (right). Veh + Pal, n = 8; IMQ + Pal, n = 8; IMQ + EPA, n = 9. Data shown are mean value ± SEM of representative experiments at least three times independently. Statistical analysis was performed with Dunn’s test (E: IgM) or Tukey’s test (if not otherwise stated). *p < 0.05; **p < 0.01; ns, not significant. See also **Figure S1**.

To elucidate the effect of EPA on the progression of SLE, we first induced lupus in wild-type FVB/N mice using imiquimod (IMQ), a TLR7 agonist, and prophylactically fed the mice a diet supplemented with 5% EPA or 5% palmitate as a control to match caloric intake (**Figure 1A** and **Table S3**). We confirmed that similar amount of palmitate intake does not affect the pathology of lupus compared to a diet without additional fatty acid (data not shown). Dietary EPA supplementation significantly increased total C20:5 (EPA) levels, whereas C20:4 (arachidonic acid) and C22:6 (docosahexaenoic acid, DHA) levels were decreased in serum, which is consistent with previous findings (**Figure 1B**) (22, 23). In addition, EPA supplementation was also linked to decreased triglyceride and cholesterol levels in serum (**Figure 1C**). These results validated the proper lipid-lowering effects of EPA. Consistent with a previous report (24), the epicutaneous application of IMQ resulted in splenomegaly, lymphadenopathy, increased deposition of immunoglobulin G (IgG) and complement component 3 (C3) in kidney glomeruli, and elevated levels of anti-nuclear (ANA), anti-double stranded DNA (dsDNA) and anti-histone autoantibodies (**Figures 1D, E** and **Figure S1**). There was no difference in body weight between the groups, suggesting that neither IMQ nor EPA attenuated dietary consumption (**Figure S1**). Under this condition, EPA supplementation attenuated the IMQ-induced deposition of IgG and C3 in the kidneys and suppressed the abundance of serum autoantibodies that are typical lupus pathologies, however, total IgG and IgM levels in serum were unchanged (**Figures 1D, E**), indicating that EPA ameliorates autoimmunity but not general antibody production. The type I interferons (IFN-I), IFNα and IFNβ, have been linked to TLR7-induced autoantibody production and systemic autoimmunity (24). Indeed, serum IFNα/β levels were increased in the IMQ-induced lupus model, and their elevation was suppressed by EPA supplementation (**Figure 1F**). White pulp in spleen is a region in which naïve T cells are activated in response to signals from APCs and B cells, and it is associated with B cell maturation and antibody production. IMQ-treated mice exhibited an enlarged white pulp region in the spleen compared with the findings in control vehicle-treated mice, and EPA supplementation attenuated the enlargement of white pulp (**Figure 1G**), indicating that the immune cell frequency and cellularity in spleen are affected by IMQ treatment and/or EPA supplementation.

### EPA Reduces Plasma Cell Counts in the Spleen of IMQ-Induced Lupus Mice

**Figure 2:**
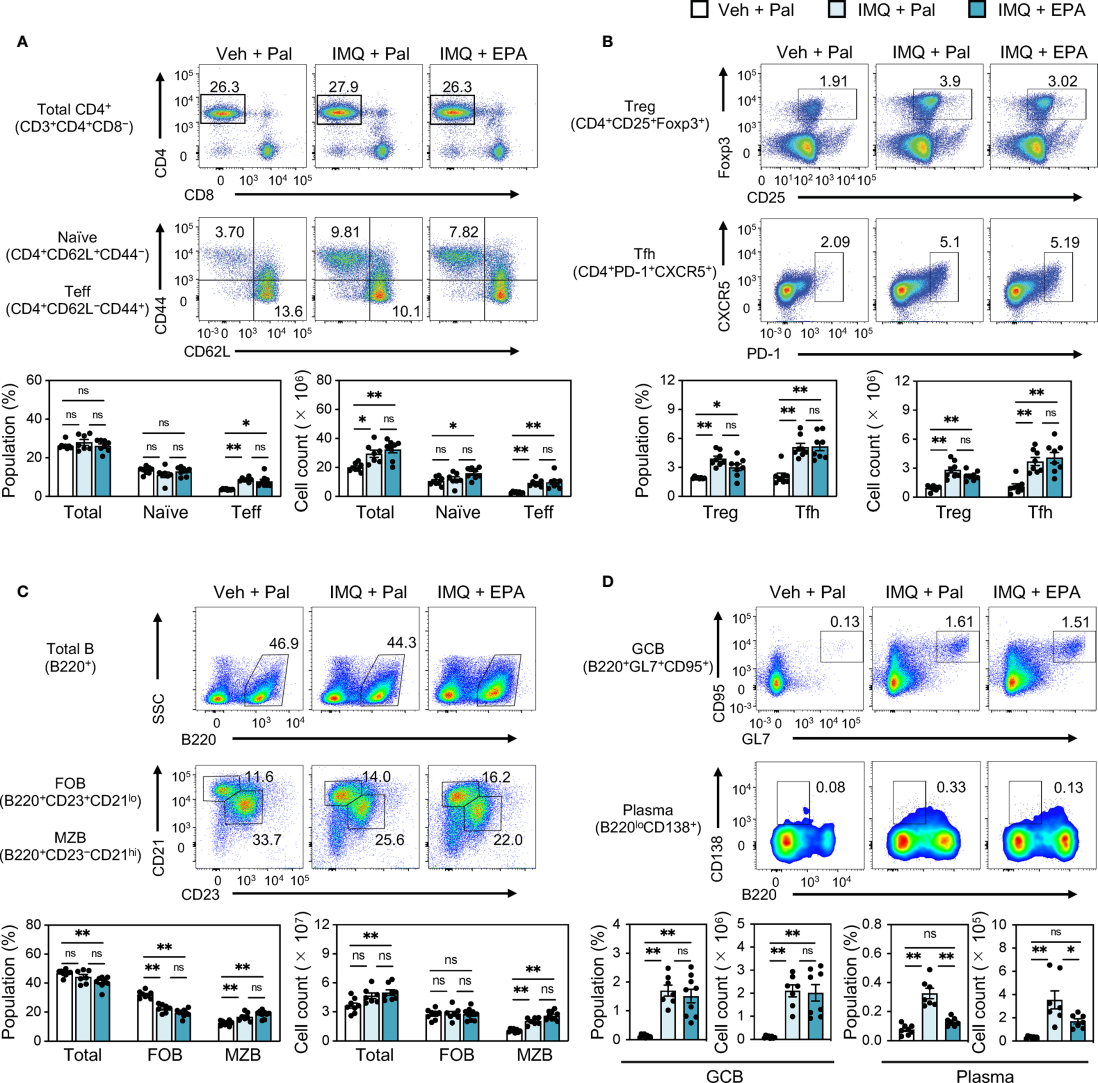
EPA reduces plasma cell counts in spleen of IMQ-induced lupus mice. Immunophenotyping of spleen from Veh-treated mice fed Pal-supplemented diet, IMQtreated mice fed Pal-supplemented diet, and IMQ-treated mice fed EPA-supplemented diet by flow cytometry. Gating strategy is available in **Figure S2**. **(A, B)** Representative flow cytometry plots (top), percentages (bottom, left) and cell counts (bottom, right) of CD4^+^ T cell subsets in total spleen cells. Total CD3^+^CD4^+^ T cells, CD4^+^ CD62L^+^ CD44^−^ naïve T cells and CD4^+^CD62L^−^CD44^+^ effector T cells (Teff) **(A)**, CD4^+^CD25^+^Foxp3^+^ regulatory T cells (Treg) and CD4^+^PD-1^+^CXCR5^+^ follicular helper T cells (Tfh) **(B)** were analyzed. **(C)** Representative flow cytometry plots (top), percentages (bottom, left) and cell counts (bottom, right) of B220^+^ B cell subsets in total spleen cells. Total B220^+^ B cells, B220^+^CD23^+^CD21^lo^ follicular B cells (FOB), B220^+^CD23^−^CD21hi marginal zone B cells (MZB) were analyzed. **(D)** Representative flow cytometry plots (top), percentages and cell counts (bottom) of B cell subsets in total spleen cells. B220^+^GL7^+^CD95^+^ germinal center B cells (GCB) and B220^lo^CD138^+^ plasma cells (Plasma) were analyzed. Veh + Pal, n = 8; IMQ + Pal, n = 8; IMQ + EPA, n = 9. Data shown are mean value ± SEM of representative experiments at least three times independently. Statistical analysis was performed with Dunn’s test (**A**: Teff) or Tukey’s test (if not otherwise stated). *p < 0.05; **p < 0.01; ns, not significant. See also **Figure S2** and **S3**.

To clarify what cell types are influenced by IMQ treatment and/or EPA supplementation, we next performed immunophenotyping of spleens using flow cytometry (**Figure S2**). Despite altered IFN-I levels (**Figure 1F**), the absolute counts of its main producers, namely dendritic cells (DCs) and plasmacytoid DCs (pDCs), were not altered by IMQ treatment or EPA supplementation (**Figure S3A**). In addition, there was no difference in the proportions and absolute numbers of monocytes and neutrophils that are mainly localized in the red pulp region among the three groups (**Figure S3A**). Regarding effector memory and follicular helper CD4 T cells and regulatory T cells, their percentages and absolute counts in the spleen were increased by IMQ treatment, whereas EPA had no effect on any T cell subset (**Figures 2A, B** and **Figure S3B**). Although there was no difference in total and follicular B cell counts among the groups, we observed increased in the populations and absolute numbers of marginal zone (MZ) B cells, germinal center (GC) B cells and plasma cells in IMQ-treated mice, and these changes are considered to reflect the vigorous immune response (**Figures 2C, D**). Remarkably, the populations and counts of plasma cells, but not MZ or GC B cells, were diminished by EPA supplementation (**Figures 2C, D**). Collectively, the data in **Figures 1** and **2** suggest that EPA attenuates IMQ-induced systemic lupus-related pathology by suppressing IFN-I production and plasma cell differentiation without affecting B cell differentiation into MZ B cells or GC B cells.

### EPA Supplementation Ameliorates Autoimmunity in C57BL/6J*^lpr/lpr^* Spontaneous Lupus Mice

**Figure 3:**
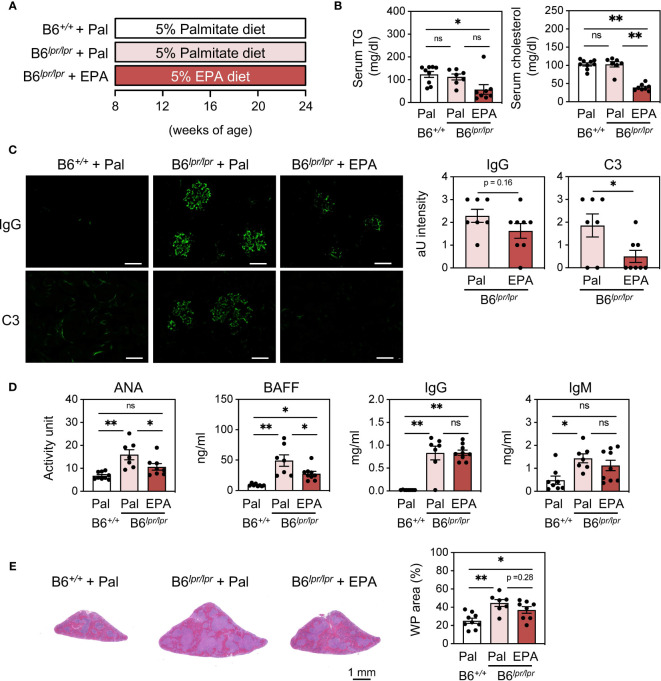
EPA supplementation ameliorates autoimmunity in C57BL/6J*^lpr/lpr^* spontaneous lupus mice. **(A)** Experimental protocol. Eight week-old control C57BL/6J*^+/+^* and C57BL/6J*^lpr/lpr^* mice were fed CE-2 diet supplemented with 5% (*wt/wt*) Pal or EPA for 16 weeks. **(B)** Serum TG and total cholesterol levels. **(C)** Immunostaining of FITC-labeled anti-mouse IgG or C3 in kidney (left) and quantification of IgG and C3 deposition (right). Original magnification: × 400, Scale bars represent 50 μm. **(D)** Serum levels of ANA, B cell activating factor (BAFF), total IgG and IgM. **(E)** H&E staining of spleen sections (left) and quantification of WP area in whole spleen area (right). B6*^+/+^* + Pal, n = 9; B6*^lpr/lpr^* + Pal, n = 7; B6*^lpr/lpr^* + EPA, n = 8. Data shown are mean value ± SEM of representative experiments at least twice independently. Statistical analysis was performed with Dunn’s test (**B, D**: IgG) or Tukey’s test (if not otherwise stated). *p < 0.05; **p < 0.01; ns, not significant. See also **Figure S4**.

**Figure 4:**
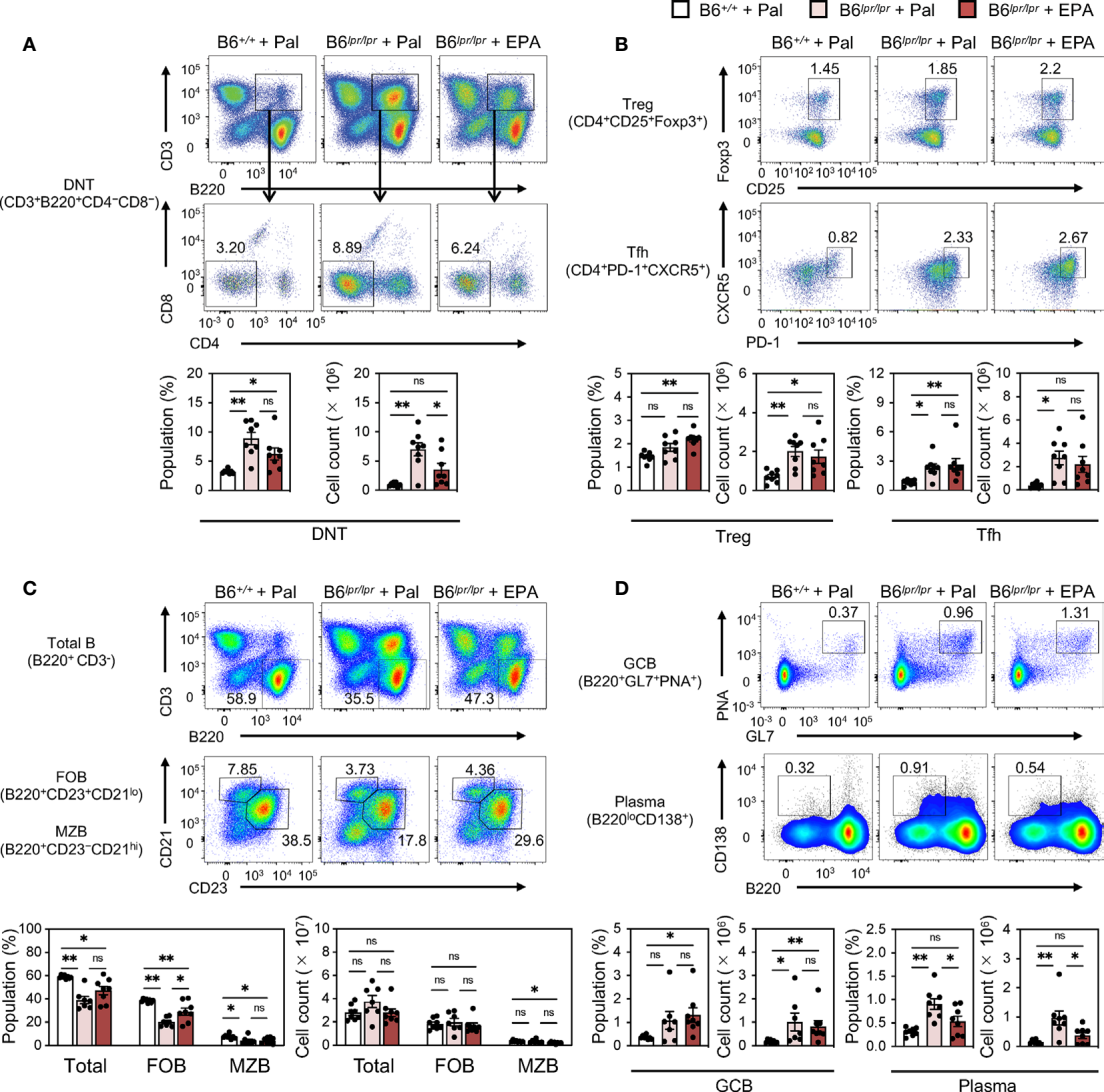
EPA reduces plasma cells in spleen of spontaneous lupus mice. Immunophenotyping of spleen from B6^+/+^ mice fed Pal-supplemented diet, B6^*lpr/lpr*^ mice fed Pal-supplemented diet, and B6^*lpr/lpr*^ mice fed EPA-supplemented diet by flow cytometry. Gating strategy is available in **Figure S5**. **(A)** Representative flow cytometry plots (top), percentages (bottom, left) and cell counts (bottom, right) of CD3^+^B220^+^CD4^−^CD8^−^ double negative T cells (DNT) in total spleen cells. **(B)** Representative flow cytometry plots (top), percentages and cell counts (bottom) of CD4^+^ T cell subsets in total spleen cells. CD4^+^CD25^+^Foxp3^+^ regulatory T cells (Treg) and CD4^+^PD-1^+^CXCR5^+^ follicular helper T cells (Tfh) were analyzed. **(C)** Representative flow cytometry plots (top), percentages (bottom, left) and cell counts (bottom, right) of B220^+^ B cell subsets in total spleen cells. Total B220^+^CD3^-^ B cells, B220^+^CD23^+^CD21^lo^ follicular B cells (FOB), B220^+^CD23^−^CD21^hi^ marginal zone B cells (MZB) were analyzed. **(D)** Representative flow cytometry plots (top), percentages and cell counts (bottom) of B cell subsets in total spleen cells. B220^+^GL7^+^CD95^+^ germinal center B cells (GCB) and B220^lo^CD138^+^ plasma cells (Plasma) were analyzed. B6^+/+^ + Pal, n = 9 ; B6^*lpr/lpr*^ + Pal, n = 7 ; B6^*lpr/lpr*^ + EPA, n = 8. Data shown are mean value ± SEM of representative experiments at least twice independently. Statistical analysis was performed with Dunn’s test (**D**: GCB) or Tukey’s test (if not otherwise stated). *p < 0.05; **p < 0.01; ns, not significant. See also **Figure S5** and **S6**.

To further assess the effect of EPA on autoimmunity in the setting of chronic spontaneous lupus, we employed C57BL/6J*^lpr/lpr^* (B6*^lpr/lpr^*) mice, which carry a mutation in the apoptosis-inducing receptor gene *Fas* and develop lupus manifestations as early as 8 week-old (25) (**Figure 3A**). Similarly as observed in IMQ-induced mice, EPA supplementation lowered serum triglyceride and cholesterol levels in B6*^lpr/lpr^* mice (**Figure 3B**). By 24 weeks of age, B6*^lpr/lpr^* mice developed profound splenomegaly and lymphadenopathy (**Figure S4**). In addition, liver weight was also increased in B6*^lpr/lpr^* mice, and hence increased body weight (**Figure S4B**), and accordingly, they exhibited enhanced deposition of immunocomplexes in the glomeruli, increased ANA levels, and white pulp enlargement (**Figures 3C–E**). This mouse model does not exhibit interferon signature, but increases production of BAFF, a critical factor supporting the survival and differentiation of B cells and a therapeutic target for SLE. Indeed, BAFF production was significantly increased in the serum of B6*^lpr/lpr^* mice (**Figure 3D**). All of these pathologies of lupus, but not increased levels of total IgG and IgM in B6*^lpr/lpr^* mice, were attenuated by EPA supplementation (**Figures 3C–E**). Immunophenotyping of spleens from B6*^lpr/lpr^* mice revealed that EPA strongly and specifically reduced the proportion and absolute number of plasma cells (**Figures 4A–D** and **Figures S5**, **S6**). Consistent with previous findings in *Fas* mutant mice (26), the induction of double-negative (CD4^−^CD8^−^) CD3^+^B220^+^ abnormal T cells in B6*^lpr/lpr^* mice was robust, and the T lymphoid cellularity was accordingly affected (**Figure 4A**). However, EPA exerted minimal effects on T lymphoid, B lymphoid, and myeloid cellularity excluding plasma cells (**Figures 4B−D** and **Figures S5, S6**). Taken together, these results suggest that EPA supplementation ameliorates autoimmunity in multiple lupus mouse models by suppressing inflammatory cytokine production and the differentiation of naïve B cells to plasma cells.

### EPA Suppresses Inflammatory Cytokine Production in CD11c^+^ Dendritic Cells

**Figure 5:**
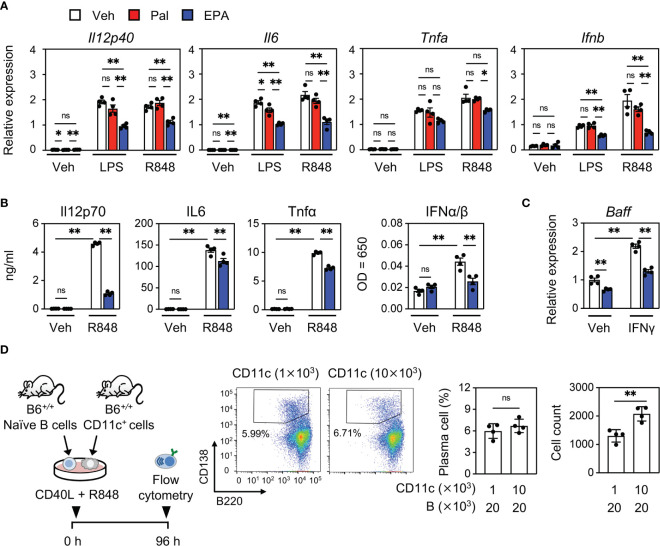
EPA suppresses inflammatory cytokine production in CD11c^+^ dendritic cells. **(A, B)** Gene expression **(A)** and cytokine levels in culture medium **(B)**. Bone marrow cells from B6*^+/+^* mice were differentiated to dendritic cells. Bone marrow–derived dendritic cells (BMDCs) were treated with ethanol (Veh), Pal (50 µM) or EPA (50 µM) for overnight, followed by stimulation with PBS (Veh), LPS (10 ng/ml) or R848 (1 μg/ml) for 4 h. **(C)** Gene expression of *Baff*. BMDCs were treated with ethanol (Veh) or EPA (50 µM) for overnight, followed by stimulation with recombinant murine IFNγ (5 ng/ml) for 6 h. **(D)** Experimental protocol (left), representative flow cytometry plots (middle), and percentages and cell counts of CD138^+^B220^lo^ plasma cells in live cells (right) analyzed by flow cytometry. Naïve B cells and CD11c^+^ cells isolated from B6*^+/+^* spleen were co-cultured at indicated cell numbers in the presence of CD40L (1 μg/ml) and R848 (1 μg/ml) for 96 h. n = 4 per group. Data shown are mean value ± SEM of representative experiments at least three times independently. Statistical analysis was performed with Turkey’s test **(A–C)** and unpaired Student’s *t* test **(D)**. *p < 0.05; **p < 0.01; ns, not significant.

Our previous report revealed that the dysregulation of lipid homeostasis in CD11c^+^ cells is a cause of increased cytokine production and systemic autoimmunity (14). Together with our observations that EPA supplementation blunts the production of cytokines that are mainly produced by CD11c^+^ cells, we speculated that the anti-inflammatory effect of EPA in CD11c^+^ cells is a primary mechanism of its action. In support of this hypothesis, EPA, but not palmitate suppressed the expression and production of inflammatory cytokines, including IFN-I, induced by the TLR7 ligand R848 in bone marrow−derived dendritic cells (BMDCs, **Figures 5A, B**). We also confirmed a previous report in which EPA suppresses TLR4 ligand lipopolysaccharide (LPS)-induced inflammatory changes (**Figures 5A, B**) (27). On the other hand, TLR4- or TLR7-induced inflammatory changes were not affected by palmitate (**Figures 5A, B**). EPA also suppressed IFNγ-induced *Baff* expression (**Figure 5C**). To further examine whether cytokines produced by CD11c^+^ cells drive the expansion of autoreactive B cells, we cocultured naïve B cells with different numbers of CD11c^+^ cells in the presence of CD40 ligand (CD40L) and R848. Naïve B cells were differentiated into B220^lo^CD138^+^ plasma cells in the presence of CD40L and R848, and the induction of plasma cells was further enhanced by coculture with larger numbers of CD11c^+^ cells (**Figure 5D**). These results indicate that EPA suppresses the production of inflammatory cytokines by CD11c^+^ dendritic cells, which contributes to inhibiting B cell differentiation into plasma cells.

### EPA Directly Inhibits Plasma Cell Differentiation

**Figure 6:**
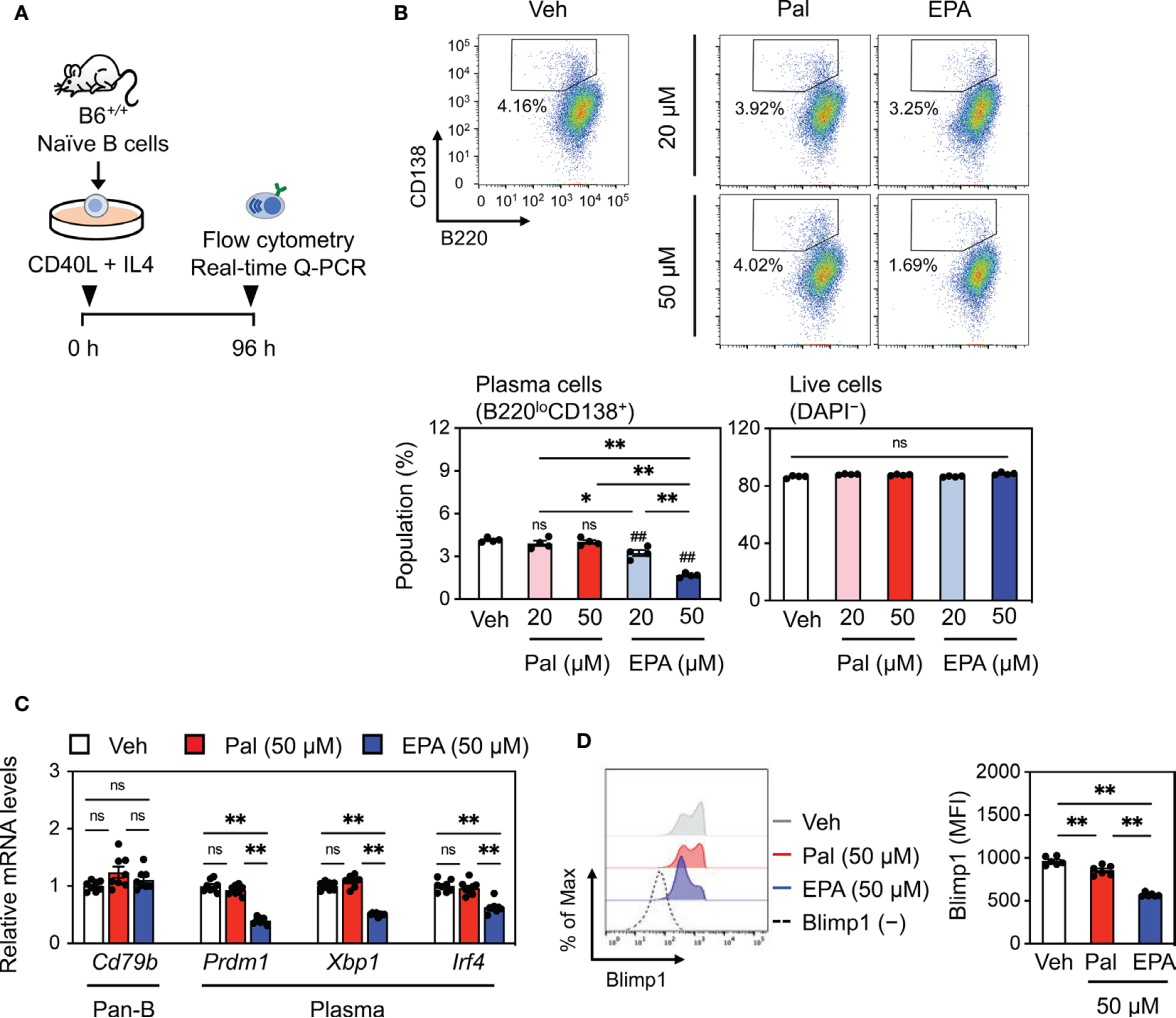
EPA directly inhibits plasma cell differentiation. **(A)** Experimental protocol. Naïve B cells isolated from B6*^+/+^* spleen were stimulated with CD40L (1 μg/ml) and recombinant murine interleukin-4 (IL4, 3 ng/ml) in the presence of ethanol (Veh), Pal (~50 µM) or EPA (~50 µM) for 96 h. **(B)** Representative flow cytometry plots (top) and percentages of B220^lo^CD138^+^ plasma cells (bottom, left) and live cells (bottom, right) analyzed by flow cytometry. **(C)** Gene expression of B cell markers. **(D)** Representative histogram (left) and mean fluorescence intensity (MFI, right) of Blimp1 in live cells. n = 4 per group. Data shown are mean value ± SEM of representative experiments at least three times independently. Statistical analysis was performed with Tukey’s test. *p < 0.05; **p < 0.01; ns, not significant.

Although a few studies focused on the mechanism by which EPA influences B cell development in the bone marrow and cytokine production in B cells (28, 29), the effects of EPA on the differentiation of naïve B cells into plasma cells and its effect on cell function and antibody production remain largely unexplored. We induced naïve wild-type splenic B cells to undergo plasma cell differentiation *via* treatment with CD40L and recombinant murine interleukin-4 (IL4) in the presence of vehicle, palmitate, or EPA (**Figure 6A**). Notably, EPA concentration-dependently diminished the plasma cell population, whereas palmitate had no effect (**Figure 6B**). Under this condition, both EPA and palmitate did not affect B cell viability (**Figure 6B**). Moreover, EPA suppressed the expression of *Prdm1* (encoding B lymphocyte-induced maturation protein-1, Blimp1)*, Xbp-1*, and *Irf4*, which are essential transcription factors for plasma cell differentiation, whereas it did not affect the expression of *Cd79b*, a transmembrane protein that forms a complex with the B cell receptor (BCR) and that is expressed in all B lineage cells (**Figure 6C**). In particular, increased Blimp1 expression in patients with SLE or lupus mouse models is associated with the number of plasma cells, abundance of autoantibodies, and disease activity (30). In line with this finding, EPA also suppressed Blimp1 protein expression (**Figure 6D**). In contrast, palmitate did not show these effects (**Figures 6B–D**). Collectively, these data demonstrate that EPA directly inhibits plasma cell differentiation by suppressing Blimp1 expression without affecting B cell viability.

### EPA Modulates Lipid Composition and Dynamics of Cellular Membrane in B Cells

**Figure 7:**
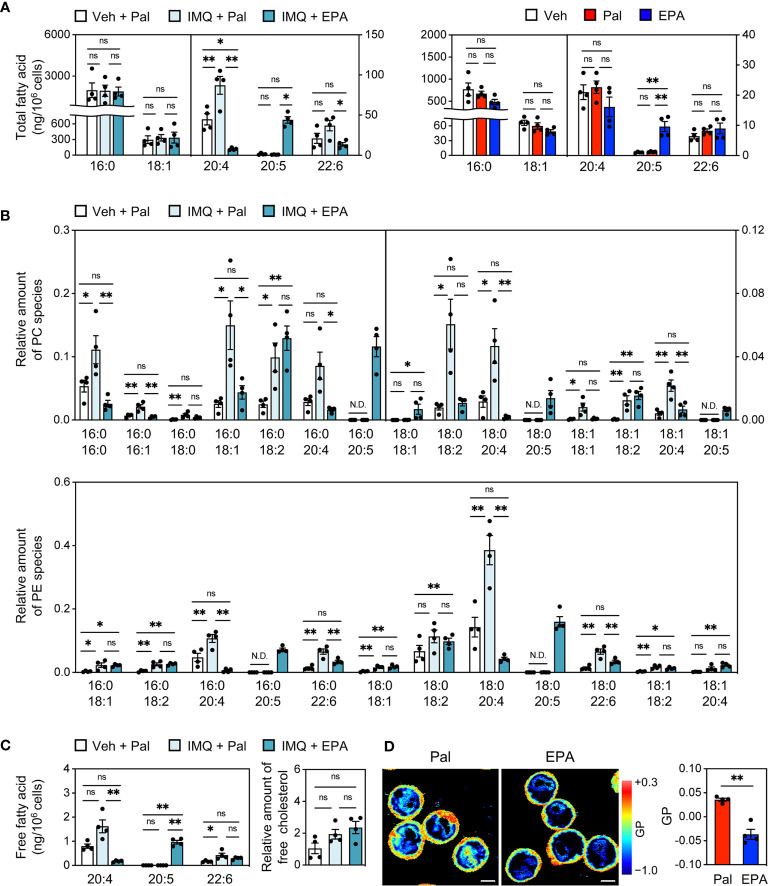
EPA modulates lipid composition and dynamics of cellular membrane in B cells. **(A)** GC-MS analysis of total fatty acid in pan-B cells of spleen from Veh-treated mice fed Pal-supplemented diet, IMQ-treated mice fed Pal-supplemented diet, and IMQ-treated mice fed EPA-supplemented diet (left) and naïve B cells isolated from B6*^+/+^* spleen stimulated with CD40L (1 μg/ml) and IL4 (3 ng/ml) in the presence of ethanol (Veh), Pal (50 μM) or EPA (50 μM) overnight (right). **(B, C)** LC-MS/MS analysis of phosphatidylcholine (PC) species (B, top), phosphatidylethanolamine (PE) species (**B**, bottom), free fatty acid (**C**, left) and free cholesterol (**C**, right) in pan-B cells of spleen from Veh-treated mice fed Pal-supplemented diet, IMQ-treated mice fed Pal-supplemented diet, and IMQ-treated mice fed EPA-supplemented diet. **(D)** Laurdan general polarization (GP) images of Ba/F3 cells (left) and quantified GP value of whole cells (right). Ba/F3 cells were treated with Pal (100 μM) 8 h then replaced by medium containing of Pal (100 μM) or EPA (100 μM) and incubated overnight. The GP value of each pixel was used to generate pseudocolored GP image. Higher GP value indicates more ordered and less dynamic membrane. Original magnification: × 600, Scale bars represent 5 μm. n = 4 per group. Data shown are mean value ± SEM. Statistical analysis was performed with Dunn’s test (**A** (left): 20:5, **B** (top): 16:0/20:4, 18:0/18:2, 18:1/18:1, **C**: 20:4), unpaired Student’s *t* test **(D)** or Tukey’s test (if not otherwise stated). *p < 0.05; **p < 0.01; ns, not significant; N.D., not detected.

**Figure 8:**
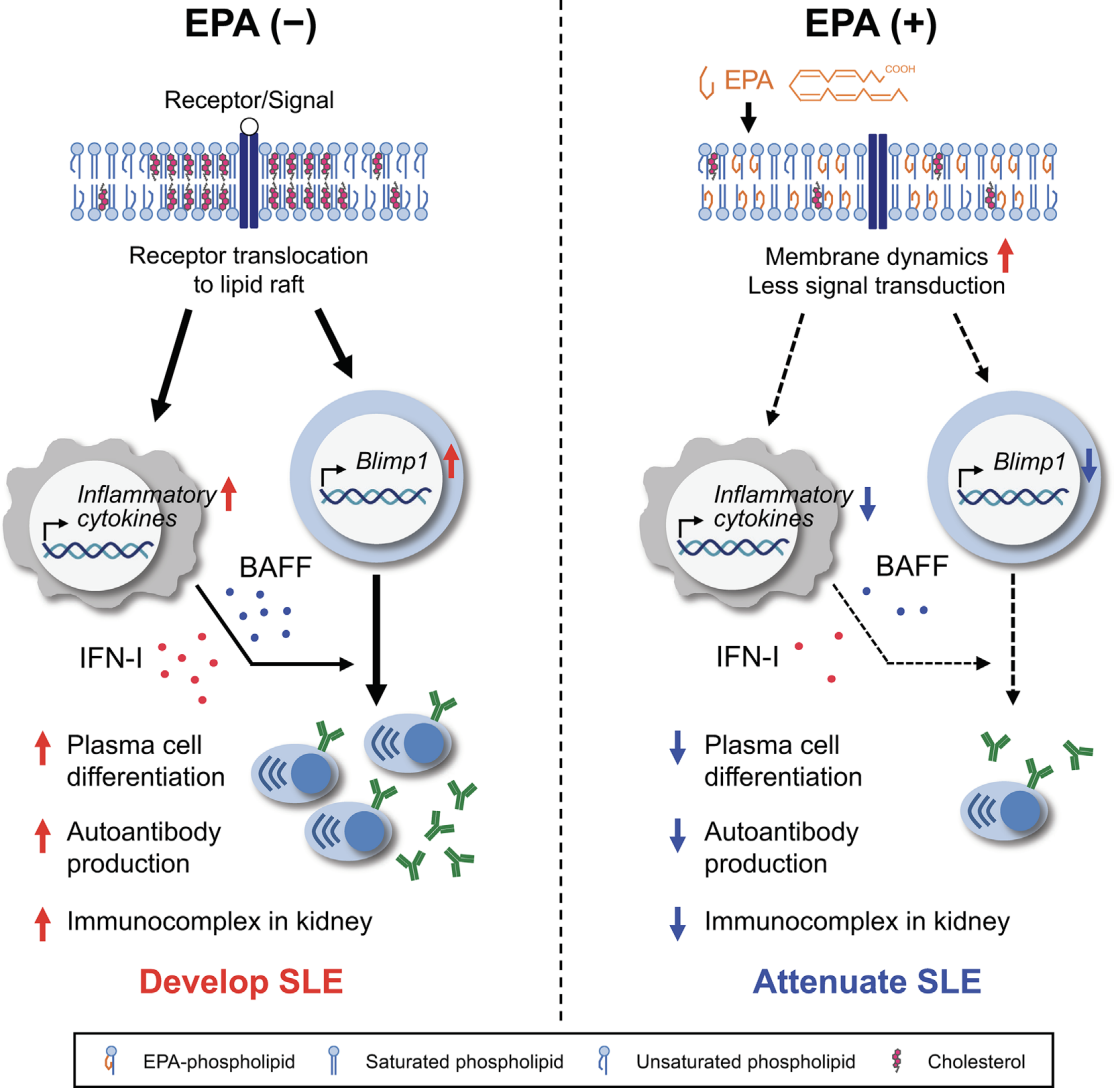
Graphical abstract. Systemic lupus erythematosus (SLE) is a chronic systemic autoimmune disease that is characterized by autoantibody-producing plasma cell differentiation, autoantibody production and immunocomplex deposition in kidney. Production of IFN-I and/or BAFF by antigen-presenting cells (APCs) have been linked to autoantibody production and the pathogenesis of SLE (left). Dietary EPA supplementation ameliorated representative SLE manifestations. EPA suppresses the production of IFN-I and BAFF by APCs. In addition, EPA remodels the lipid composition and increases membrane fluidity in B cells, thereby preventing the signal for plasma cell differentiation (right). This study highlights a mechanism by which cellular fatty acids regulates the B cell function in the context of systemic autoimmunity.

Finally, we investigated the potential mechanisms underlying the suppression of plasma cell differentiation by EPA. The molecular mechanisms of EPA are greatly pleiotropic (8, 9). Among others, based on our previous findings that increased abundance of cholesterol and polyunsaturated phospholipids in cells enhances their membrane dynamics (18, 19, 31), we hypothesized that EPA alters the cellular lipid composition in B cells, consequently resulting in dynamic cellular membranes. To test this speculation, we performed cellular lipid analysis using splenic pan-B cells from IMQ-induced lupus mice that were supplemented with EPA or control palmitate. Similar to the changes in their serum levels, C20:5 levels in total fatty acid was substantially increased while C20:4 and C22:6 levels were decreased in pan-B cells after dietary EPA intake (**Figure 7A**, left). Increased C20:5 content was also observed in isolated splenic pan-B cells that were directly treated with EPA (**Figure 7A**, right). These data suggest that EPA is incorporated into B cells. Global analysis of phospholipids by liquid chromatography−tandem mass spectrometry (LC-MS/MS) revealed that the amount of C20:5 levels in phosphatidylcholine (PC) and phosphatidylethanolamine (PE) were profoundly increased. The levels of most PC- and PE-containing moieties, excluding C20:5- and C18:2 (linoleic acid)-containing phospholipids, were decreased following EPA treatment, but the rate of the decrease was much smaller than the rate of increase of C20:5 (**Figure 7B**). Additionally, free C20:5 levels in B cells were markedly increased (**Figure 7C**). Free EPA is considered to be elicited from phospholipids by phospholipase A2; however, the levels of lysoPC and lysoPE were not altered by EPA supplementation (data not shown). Thus, EPA is incorporated into cells as a free fatty acid to some extent. Although a compensatory increase of cholesterol levels has been reported in DHA-treated cells to maintain the membrane homeostasis (32), there was no change in the amount of free cholesterol in our study (**Figure 7C**). These findings led us to speculate that dietary EPA supplementation makes cellular membranes less rigid. In fact, laurdan generalized polarization (GP) was profoundly decreased in EPA-treated Ba/F3 cells, which is indicative of membranes with increased lipid mobility (**Figure 7D**). Taken together, these results suggest that dietary EPA remodels the membrane lipid composition and thereby increases membrane dynamics in B cells, which in turn suppresses downstream signaling for plasma cell differentiation and autoantibody production (**Figure 8**).

## Discussion

Although EPA exerts beneficial effects against inflammatory diseases, its effects on adaptive immune responses has been less extensively studied, and little is known about the mechanism by which EPA influences autoimmunity, especially B cell differentiation and autoantibody production. In this study, we have demonstrated that dietary EPA suppresses B cell differentiation into autoantibody-producing plasma cells, enhances anti-inflammation in CD11c+ dendritic cells and attenuated the pathology of lupus in two mouse models. Recent studies have drawn attention to plasma cells as therapeutic targets in autoimmune diseases. Depletion of autoreactive plasma cells in lupus mouse models prevents autoantibody production and the development of lupus nephritis (33–35). Contrarily, the adoptive transfer of plasma cells from lupus-prone mice to the mice lacking mature lymphocytes induces autoantibody production (36). Hence, eliminating plasma cells or blocking the production of autoantibodies has been considered an efficient treatment strategy for SLE. Indeed, belimumab, an anti-BAFF human monoclonal antibody, has been approved for treatment of SLE. However, at least 40% of patients with SLE did not display a clinically meaningful response to belimumab, suggesting the molecular heterogeneity of SLE. Our findings that EPA directly regulates plasma cell differentiation, which is attributed in part to the altered abundance of cellular fatty acids and increased membrane fluidity, highlight a previously unrecognized role of EPA in autoimmunity. EPA has been reported to have beneficial roles not only in SLE but also in other autoimmune diseases, such as rheumatoid arthritis, multiple sclerosis and type 1 diabetes (10). Further insight into lipid metabolism and lipid composition in B cell membrane on the production of autoantibodies would shed light on understanding a general mechanism for autoimmune diseases.

Lipid rafts are cholesterol- and sphingolipid-enriched membrane microdomains that are considered to function as membrane signaling platforms. We and others previously reported that decreased cholesterol content or elevated polyunsaturated PC content in cellular membranes resulted in increased fluidity, thereby inhibiting the recruitment of inflammatory signaling pathways downstream of membrane-bound c-Src kinase or membrane receptors, such as TLR2, TLR4 and TLR9 (18, 19, 31). Whereas omega-3 fatty acids have already been reported to have marked effects on membrane order (32, 37, 38), our lipidomic and laurdan GP data newly illustrated that profound increases in polyunsaturated EPA-containing phospholipid and free EPA levels without changes in cholesterol content in B cells result in the formation of flexible and fluid membranes. Consequently, the dynamic alteration of the membrane would disrupt the translocation of CD40 receptor or BCR to lipid rafts, thereby preventing the downstream activation of NF-κB and the transactivation of *Prdm1*/Blimp1, which is induced by NF-κB (39). In fact, our *in vitro* B cell differentiation assay demonstrated that EPA suppresses Blimp1 expression at both the transcriptional and translational levels. It also has been reported that BAFF/BAFF receptor binding prolongs the association of BCR and lipid rafts (40). Therefore, EPA likely suppresses the signaling for plasma cell differentiation by increasing membrane flexibility in coordination with reduced BAFF production by dendritic cells (**Figure 8**).

In addition to altering membrane lipid composition in B cells, it is possible that EPA directly modifies B cell function *via* G-protein coupled receptor (GPR120), a potent receptor for omega-3 fatty acids including EPA, and/or pro-resolving lipid mediators, *e.g.*, resolvin E1 and E2, derived from EPA and their receptors (41, 42). For instance, a recent report demonstrated that a pro-resolving mediator of DHA enhances antibody production against influenza virus (43). Although our lipidomic analysis did not cover EPA metabolites, we assume that the levels of the pro-resolving metabolites of EPA in serum or tissue microenvironment were increased by dietary EPA supplementation. Besides, we need to elucidate the role of palmitate included in the control diet in the pathogenesis of lupus autoimmunity, along with the fatty acid composition, membrane dynamics, and cell function of immune cells. Collectively, the primary site of action of EPA is still unclear, and further studies are needed to elucidate the molecular mechanism underlying EPA-mediated amelioration of lupus autoimmunity.

In this study, we demonstrated that reduced cytokine production by CD11c^+^ dendritic cells following EPA treatment also contributes to prevent plasma cell differentiation. In particular, EPA suppressed the production of IFN-I and BAFF, which are known to regulate plasma cell differentiation and play critical roles in the pathology of lupus (44). According to previous reports (18, 19, 31), this anti-inflammatory effect of EPA in dendritic cells is attributable at least in part to its effect on physiological properties of membranes. It is also possible that the anti-inflammatory effect of EPA is mediated by direct binding to GPR120 and/or pro-resolving lipid mediators and their receptors (41, 42). Indeed, CD11c^+^ dendritic cells highly express these receptors. In addition, we observed the reduced arachidonic acid levels following EPA supplementation, which may reduce the levels of pro-inflammatory eicosanoids (prostaglandinds, thromboxanes and leukotrienes) and contribute to the prevention of inflammation (45).

One of the major concerns about current immunosuppressive treatments or B cell-target therapies for autoimmune diseases is the increased risk of infection. Our finding that EPA suppresses plasma cell differentiation and autoantibody production raises questions concerning whether EPA increases the susceptibility to infection and deteriorates vaccine efficacy because of defective antibody production. Despite being sparse, previous studies demonstrated that dietary EPA intake does not influence immunoglobulin production in response to LPS or salmon vaccine or the clearance of influenza virus (46, 47). We also revealed in this study that dietary EPA supplementation does not decrease total IgG and IgM levels in mice with lupus. Shaikh et al. reported in the context of obesity and chronic systemic inflammation that dietary intake of fish oil, a mixture of EPA and DHA, restores impaired antibody production to a T cell-independent antigen (28). Additional studies are needed to clarify the mechanism by which EPA influences antibody production during infection or vaccination. Considering that EPA is beneficial for ameliorating infection (8, 48), it is less likely that EPA worsens the antibody production in response to infectious antigens.

Recent longitudinal transcriptomic data indicated that patients with SLE can be stratified into multiple groups according to their molecular blood signatures, *e.g.*, IFN-I signature and BAFF signature. These findings may help to explain the failure of drugs targeting CD20 and IFNα in clinical trials and the diverse efficacy of anti-BAFF treatment (49, 50). In this study, we employed two different lupus models: (1) IMQ-induced lupus, which is mediated in part by IFN-I production, to mimic patients with SLE and IFN-I signature and (2) spontaneous C57BL/6J*^lpr/lpr^* mice that exhibit BAFF, but not IFN-I production to mimic patients with SLE and BAFF signature. We demonstrated that EPA had beneficial effect in both models. Because EPA is approved for the treatment of hypertriglyceridemia and is also available as a dietary supplement, our results identify EPA as a potential universal agent with less toxicity for the treatment of SLE. Indeed, several clinical studies reported that dietary supplementation of EPA or fish oil may reduce the disease activity of SLE or prolong the remission period (10). For the next step, it is interesting to verify the effectiveness of the combination therapy of conventional medicines and EPA.

## Data Availability Statement

The original contributions presented in the study are included in the article/**Supplementary Material**. Further inquiries can be directed to the corresponding authors.

## Ethics Statement

The animal study was reviewed and approved by Committee on the Ethics of Animal Experiments of Nagoya University.

## Author Contributions

AK designed and performed experiments and wrote the manuscript. AI and TS conceived and designed the study, guided the interpretation of the results and the preparation of the manuscript. TS supervised the study and AI performed and managed the daily experiments and supervised the study. IS performed histology experiments. AT, ST, and HS performed the mass spectrometry analysis. PH supervised membrane dynamics analysis. MT, NT, TI, SA-T, and SM analyzed the data and contributed to discussion. All authors contributed to the article and approved the submitted version.

## Funding

This work was supported in part by Grants-in-Aid for Scientific Research from the Ministry of Education, Culture, Sports, Science and Technology of Japan (20H03447, 20H05503, and 20H04944 to TS, 19K11765 and 19KK0249 to AI) and Japan Agency for Medical Research and Development (CREST) (JP20gm1210009s0102 to TS, JP20gm0910011 to HS). This study was also supported by research grants from Smoking Research Foundation (TS), Takeda Science Foundation, Astellas Foundation for Research on Metabolic Disorders, Ono Medical Research Foundation, Mochida Memorial Foundation for Medical and Pharmaceutical Research, GSK Japan Research Grant 2018, SENSHIN Medical Research Foundation, Kao Research Council for the Study of Healthcare Science, Kowa Life Science Foundation, The Hori Sciences and Arts (AI), Aichi Kidney Foundation (AK). GSK was not involved in the study design, collection, analysis, interpretation of data, the writing of this article or the decision to submit it for publication.

## Conflict of Interest

The authors declare that the research was conducted in the absence of any commercial or financial relationships that could be construed as a potential conflict of interest.
