# Supplementary material for: Dietary Supplementation With Eicosapentaenoic Acid Inhibits Plasma Cell Differentiation and Attenuates Lupus Autoimmunity
Source: Front Immunol. 2021 Jun 15;12:650856. doi: 10.3389/fimmu.2021.650856 (PMC8240640; doi:10.3389/fimmu.2021.650856)
Supplement: Supplementary file 1 [file Presentation_1.pdf]

Title

Dietary supplementation with eicosapentaenoic acid inhibits plasma cell differentiation and attenuates lupus autoimmunity

Article type

Original Research Article

Authors/Affiliations

Azusa Kobayashi[1,2†], Ayaka Ito[1,3†*], Ibuki Shirakawa[1,3], Atsushi Tamura[4], Susumu Tomono[5], Hideo Shindou[6,7], Per Niklas Hedde[8], Miyako Tanaka[1,3], Naotake Tsuboi[9], Takuji Ishimoto[2], Sachiko Akashi-Takamura[5], Shoichi Maruyama[2], Takayoshi Suganami[1,3]*

[1] Department of Molecular Medicine and Metabolism, Research Institute of Environmental Medicine, Nagoya University, Nagoya, Japan

[2] Department of Nephrology, Nagoya University Graduate School of Medicine, Nagoya, Japan.

[3] Department of Immunometabolism, Nagoya University Graduate School of Medicine, Nagoya, Japan.

[4] Department of Organic Biomaterials, Institute of Biomaterials and Bioengineering, Tokyo Medical and Dental University (TMDU), Tokyo, Japan

[5] Department of Microbiology and Immunology, Aichi Medical University School of Medicine, Nagakute, Japan.

[6] Department of Lipid Signaling, National Center for Global Health and Medicine, Tokyo, Japan

[7] Department of Medical Lipid Science, Graduate School of Medicine, The University of Tokyo, Tokyo, Japan

[8] Laboratory of Fluorescence Dynamics, Biomedical Engineering Department, Center for Complex Biological Systems, University of California, Irvine, Irvine, CA

[9] Department of Nephrology, Fujita Health University Graduate School of Medicine, Toyoake, Japan

[†] These authors have contributed equally to this work and share first authorship

Correspondence:

Ayaka Ito: aito@riem.nagoya-u.ac.jp

Takayoshi Suganami: suganami@riem.nagoya-u.ac.jp

Figure S1 | Effect of EPA supplementation on body and tissue weights in lupus mice. Body and tissue weights. Four-week-old FVB/N mice were fed CE-2 diet supplemented with 5% (*wt/wt*) palmitate (Pal) or eicosapentaenoic acid (EPA) for 6 weeks. To induce lupus, the mice received imiquimod (IMQ) or vehicle (Veh) treatment 3 times a week during the last 4 weeks. Veh + Pal, n = 8; IMQ + Pal, n = 8; IMQ + EPA, n = 9. Data shown are mean value ± SEM of representative experiments at least three times independently. Statistical analysis was performed with Turkey's test. *p < 0.05; **p < 0.01; ns, not significant.

Figure S2 | Gating strategy used for immunophenotyping of spleens in **Figure 2**. Spleen from Veh-treated mice fed Pal-supplemented diet, IMQ-treated mice fed Pal-supplemented diet, and IMQ-treated mice fed EPA-supplemented diet were analyzed by flow cytometry.

Figure S3 | EPA does not affect $CD8^+$ T or myeloid cellurality in spleen of IMQ-induced lupus mice. Immunophenotyping of spleen from Veh-treated mice fed Pal-supplemented diet, IMQ-treated mice fed Pal-supplemented diet, and IMQ-treated mice fed EPA-supplemented diet by flow cytometry. Gating strategy is available in Figure S2. **(A)** Representative flow cytometry plots (left), percentages (right, top) and cell counts (right, bottom) of myeloid cells in total spleen cells. $CD11c^+MHCII^+$ dendritic cells (DC), $B220^+PDCA^+$ plasmacytoid DC (pDC), $CD11b^+Ly6G^-$ monocytes and $CD11b^+Ly6G^+$ neutrophils were analyzed. **(B)** Representative flow cytometry plots (left), percentages (right, top) and cell counts (right, bottom) of $CD8^+$ T cell subsets in total spleen cells. Total $CD3^+CD8^+$ T cells, $CD8^+CD62L^+CD44^-$ naïve T cells and $CD8^+CD62L^-CD44^+$ effector T cells (Teff) were analyzed. Veh + Pal, n = 8; IMQ + Pal, n = 8; IMQ + EPA, n = 8. Data shown are mean value ± SEM of representative experiments at least three times independently. Statistical analysis was performed with Dunn's test (A: cell count of DC and Monocyte, B: Teff) or Tukey's test (if not otherwise stated). *p < 0.05; **p < 0.01; ns, not significant.

Figure S4 | Effect of EPA supplementation on body and tissue weights in lupus mice. **(A)** Gross morphology of spleen (left) and axillary lymph nodes (right). **(B)** Body and tissue weights. Eight week-old control C57BL/6J$^{+/+}$ and C57BL/6J$^{lpr/lpr}$ mice were fed CE-2 diet supplemented with 5% (*wt/wt*) Pal or EPA for 16 weeks. Lymph node panel indicates the weight of 2 axillary lymph nodes. B6$^{+/+}$ + Pal, n = 9; B6$^{lpr/lpr}$ + Pal, n = 7; B6$^{lpr/lpr}$ + EPA, n = 8. Data shown are mean value ± SEM of representative experiments at least twice independently. Statistical analysis was performed with Turkey's test. *p < 0.05; **p < 0.01; ns, not significant.

Figure S5 | Gating strategy used for immunophenotyping of spleens in **Figure 4**. Spleen from B6$^{+/+}$ mice fed Pal-supplemented diet, B6$^{lpr/lpr}$ mice fed Pal-supplemented diet, and B6$^{lpr/lpr}$ mice fed EPA-supplemented diet were analyzed by flow cytometry.

Figure S6 | EPA does not affect CD8^{+} T or myeloid cellularity in spleen of spontaneous lupus mice. Immunophenotyping of spleen from B6$^{+/+}$ mice fed Pal-supplemented diet, B6$^{lpr/lpr}$ mice fed Pal-supplemented diet, and B6$^{lpr/lpr}$ mice fed EPA-supplemented diet by flow cytometry. Gating strategy is available in Figure S5. **(A)** Representative flow cytometry plots (left), percentages (right, top) and cell counts (right, bottom) of T cell subsets in total spleen cells. Total cells, CD62L^{+}CD44^{-} naïve T cells and CD62L^{-}CD44^{+} effector T cells (Teff) in CD4^{+} and CD8^{+} T cells were analyzed. **(B)** Representative flow cytometry plots (left), percentages (right, top) and cell counts (right, bottom) of myeloid cells in total spleen cells. CD11c^{+}MHCII^{+} dendritic cells (DC), B220^{+}PDCA^{+} plasmacytoid DC (pDC), CD11b^{+}Ly6G^{-} monocytes and CD11b^{+}Ly6G^{+} neutrophils were analyzed. B6$^{+/+}$ + Pal, n = 9; B6$^{lpr/lpr}$ + Pal, n = 7; B6$^{lpr/lpr}$ + EPA, n = 8. Data shown are mean value ± SEM of representative experiments at least twice independently. Statistical analysis was performed

with Dunn's test (A: Cell count of CD4 Total, Teff, CD8 naïve and Teff, B: population of pDC and Neutrophil, cell count of DC and pDC) or Tukey's test (if not otherwise stated). *p < 0.05; **p < 0.01; ns, not significant.

Table S1. List of resources used in this study

Antibody	Source	Clone	Identifier
Fluorescein (FITC) AffiniPure Goat Anti-Mouse IgG (H+L)	Jackson ImmunoResearch		Cat#115-095-003 RRID : AB_2338589
Fluorescein-Conjugated Goat IgG Fraction To Mouse Complement C3	MP Biomedicals		Cat#SKU 0855500
PE/Cy7 anti-mouse CD138 (Syndecan-1) Antibody	BioLegend	281-2	Cat#142513 RRID : AB_2562197
APC anti-mouse/human CD45R/B220 Antibody	BioLegend	RA3-6B2	Cat#103211 RRID : AB_312996
Brilliant Violet 510™ anti-mouse/human CD45R/B220 Antibody	BioLegend	RA3-6B2	Cat#103247 RRID : AB_2561394
PE anti-mouse CD95 (Fas) Antibody	BioLegend	SA367H8	Cat#152607 RRID : AB_2632903
PerCP/Cyanine5.5 anti-mouse CD23 Antibody	BioLegend	B3B4	Cat#101617 RRID : AB_2562923
APC/Cy7 anti-mouse CD21/CD35 (CR2/CR1) Antibody	BioLegend	7E9	Cat#123417 RRID : AB_1953274
APC anti-mouse/human GL7 Antigen (T and B cell Activation Marker) Antibody	BioLegend	GL7	Cat#144617 RRID : AB_2800674
PE anti-mouse CD3e	BioLegend	145-2C11	Cat#100308 RRID : AB_312673
Brilliant Violet 510™ anti-mouse CD4 Antibody	BioLegend	GK1.5	Cat#100449 RRID : AB_2564587
PE/Cy7 anti-mouse/human CD45R/B220 Antibody	BioLegend	RA3-6B2	Cat#103221 RRID : AB_313004
APC anti-mouse/human CD44 Antibody	BioLegend	IM7	Cat#103011 RRID : AB_312962
APC/Cy7 anti-mouse CD62L Antibody	BioLegend	MEL-14	Cat#104427 RRID : AB_830798
Brilliant Violet 510™ anti-mouse I-A/I-E Antibody	BioLegend	M5/114.15.2	Cat#107635 RRID : AB_2561397
PE anti-mouse CD317 (BST2, PDCA-1)	BioLegend	9.27E+02	Cat#127009 RRID : AB_1953284
PerCP anti-mouse CD11c Antibody	BioLegend	N418	Cat#117325 RRID : AB_893236
PE/Cy7 anti-mouse Ly-6G Antibody	BioLegend	1A8	Cat#127617 RRID : AB_1877262
APC/Cy7 anti-mouse/human CD11b Antibody	BioLegend	M1/70	Cat#101225 RRID : AB_830641

TruStain FcX™ (anti-mouse CD16/32) Antibody	BioLegend	93	Cat#101320<br>RRID : AB_1574975
PE/Cy7 anti-mouse CD279 (PD-1)	BioLegend	29F.1A12	Cat#135215<br>RRID : AB_10696422
PE anti-mouse Foxp3	TONBO Biosciences	3G3	Cat#50-5773-U025
APC anti-mouse CD25	BioLegend	3C7	Cat#101909<br>RRID : AB_961208
Alexa Fluor® 647 anti-mouse Blimp-1 Antibody	BioLegend	5E7	Cat#150003<br>RRID : AB_2565617
PerCP/cyanine5.5 anti-mouse CD8a Antibody	BioLegend	53-6.7	Cat#100733<br>RRID : AB_2075239
Cy3 Peanut Agglutinin (PNA)	Vector laboratories		Cat#CL-1073-1
Purified anti-mouse CD40 Antibody	BioLegend	HM40-3	Cat#102902<br>RRID : AB_312945

Chemical/Peptide/Recombinant protein	Source	Identifier
DAPI (used for nuclear staining)	BioLegend	Cat#422801
Collagenase Type IV	Worthington	Cat#CLS4
DNase I	Roche Diagnostics	Cat#11284932001
R848 Resiquimod	AdipoGen	Cat#AG-CR1-3582
LPS from E. coli O111:B4	Sigma-Aldrich	Cat#L4391-1MG
Recombinant murine IL4	Pepro Tech	Cat#214-14
Recombinant murine IFNγ	Pepro Tech	Cat#315−05
Palmitic acid BioXtra, ≥99%	Sigma-Aldrich	Cat#P5585
cis-5,8,11,14,17-Eicosapentaenoic acid sodium salt ≥99% (capillary GC)	Sigma-Aldrich	Cat#E6627-25MG
BESELNA cream 5%	Mochida Pharmaceutical	Cat#87629
Foxp3/Transcription Factor Staining Buffer Kit	TONBO Biosciences	Cat#TNB-0607-KIT
Laurdan	Cayman Chemical	Cat#19706

ELISA	Source	Identifier
Mouse Anti-Nuclear Antibodies (ANA) Total Ig ELISA Kit	Alpha Daiagnostic International	Cat#5210
Mouse BAFF/BLyS/TNFSF13B Immunoassay	R&D Systems	Cat#MBLYS0
LBIS Mouse Anti-dsDNA ELISA Kit	FUJIFILM Wako Shibayagi	Cat#AKRDD-061
Mouse Anti-Histones Ig's ELISA Kit, 96 tests,	Alpha Daiagnostic	Cat#5610

Quantitative	International	
Mouse IgG Uncoated ELISA Kit	Invitrogen	Cat#88-50400-22
Mouse IgM Uncoated ELISA Kit	Invitrogen	Cat#88-50470-22
Mouse IL-12 p70 ELISA Kit	Invitrogen	Cat#BMS6004
Mouse IL-6 Quantikine ELISA Kit	R&D Systems	Cat#M6000B
Mouse TNF-alpha Quantikine ELISA Kit	R&D Systems	Cat#MTA00B

Cell separation reagent	Source	Identifier
FcR Blocking Reagent, mouse	Miltenyi Biotec	Cat#130-092-575
B cell Isolation Kit II	Miltenyi Biotec	Cat#130-090-862
Pan B cell Isolation kit II	Miltenyi Biotec	Cat#130-104-443
CD11c microbeads Ultrapure	Miltenyi Biotec	Cat#130-108-338

Animal/Cell	Source	Identifier
Mouse: C57BL/6J+/+ females 8 weeks of age	Japan SLC, Inc	N/A
Mouse: C57BL/6Jlpr/lpr females 8 weeks of age	Japan SLC, Inc	N/A
Mouse: FVB/N females 4 weeks of age	CLEA Japan, Inc	N/A
Ba/F3-CL1	RIKEN BRC	Cat#RBC4474
Reporter cell: B16-Blue IFN-α/β cells	Invivogen	Cat#bb-ifnt1

Software	Source	Identifier
FlowJo software	BD Biosciene	https://www.flowjo.com/
SimFCS software	Laboratory for Fluorescence Dynamics	lfd.uci.edu/globals/
Prism9	GraphPad	https://www.graphpad.com/

Table S2. List of primers used to detect mRNAs

Name	Fw/Rv	Sequence
36b4	Fw	5'−GGCCCTGCACTCTCGCTTTC−3'
	Rv	5'−TGCCAGGACGCGCTTGT−3'
Baff	Fw	5'−AACAGACGCGCTTTCCAG−3'
	Rv	5'−AGGAGGAGCTGAGAGGTCTACAT−3'
Prdm1	Fw	5'−TTCTCTTGGAAAAACGTGTGGG−3'
	Rv	5'−GGAGCCGGAGCTAGACTTG−3'
Cd79b	Fw	5'−TGTTGGAATCTGCAAATGGA−3'
	Rv	5'−TAGGCTTTGGGTGATCCTTG−3'
Ifnb	Fw	5'−TGGAGCAGCTGAATGGAAAG−3'
	Rv	5'−ATCTCTTGGATGGCAAAGGC−3'
Il6	Fw	5'−GCTACCAAACTGGATATAATCAGGA−3'
	Rv	5'−CCAGGTAGCTATGGTACTCCAGAA−3'
Il12b	Fw	5'−ACATCTACCGAAGTCCAATGCA−3'
	Rv	5'−GGAATTGTAATAGCGATCCTGAGC−3'
Irf4	Fw	5'−ACCACTGGCAAGGCCCAT−3'
	Rv	5'−GCAGTCTGAGAGCGCCAAG−3'
Tnfa	Fw	5'−ACCCTCACACTCAGATCATCTTC−3'
	Rv	5'−TGGTGGTTTGCTACGACGT−3'
Xbp1	Fw	5'−GAGTCCGCAGCAGGTG−3'
	Rv	5'−GTGTCAGAGTCCATGGGA−3'

Table S3. Composition of experimental diets

Product	Fish meal−free diet (No. 012, CREA Japan)		Fish meal−free diet supplemented with 5% fatty acid (wt/wt)	
	kcal/100g	kcal%	kcal/100g	kcal%
Protein	80	23	76	21
Carbohydrate	222	65	212	58
Fat	39	11	80	22
Total	341	100	367	100
