# Supplementary figures and images for: Dietary Supplementation With Eicosapentaenoic Acid Inhibits Plasma Cell Differentiation and Attenuates Lupus Autoimmunity
Source: Front Immunol. 2021 Jun 15;12:650856. doi: 10.3389/fimmu.2021.650856 (PMC8240640; doi:10.3389/fimmu.2021.650856)

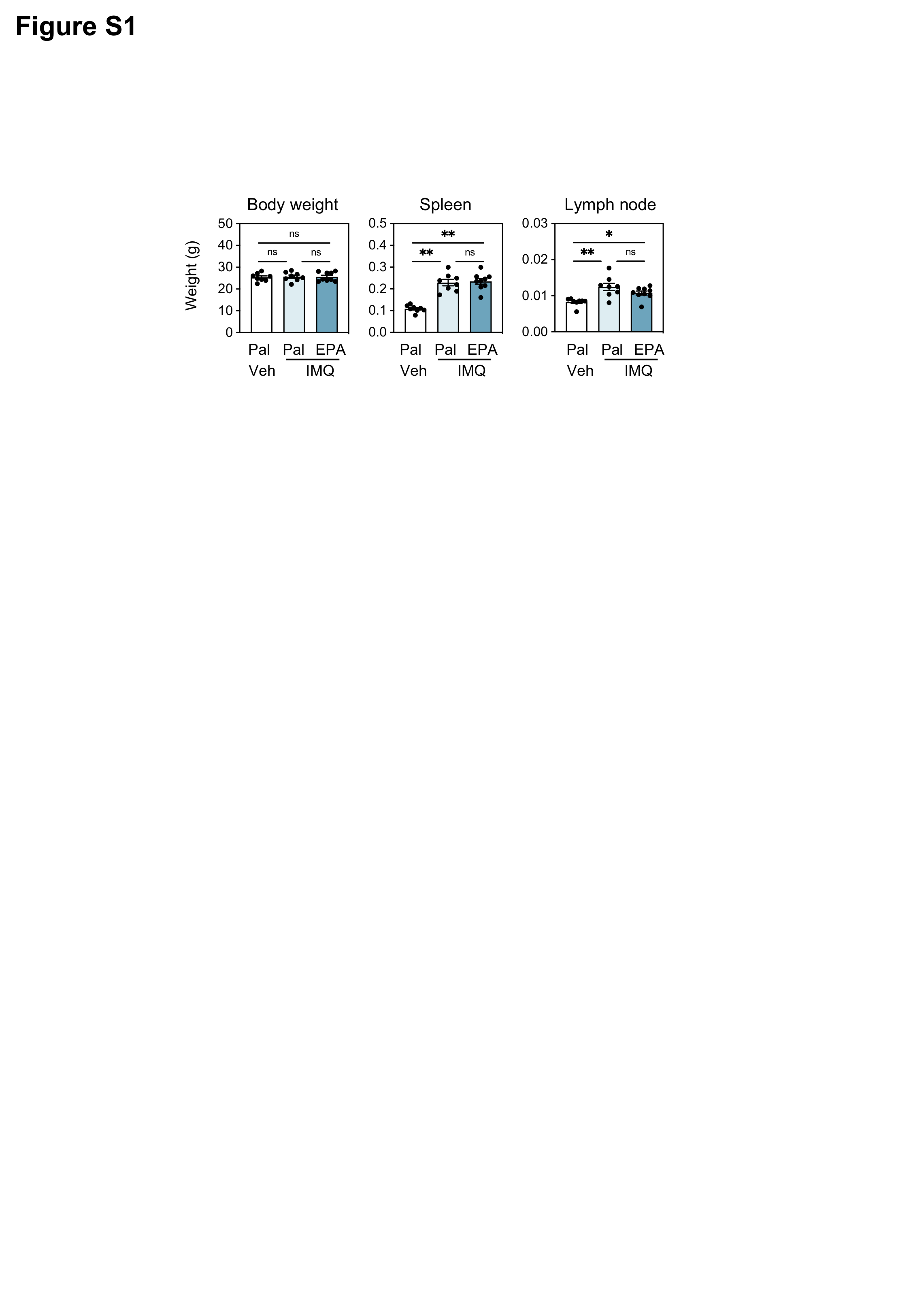

Supplement: Supplementary file 2 [file Image_1.tiff]

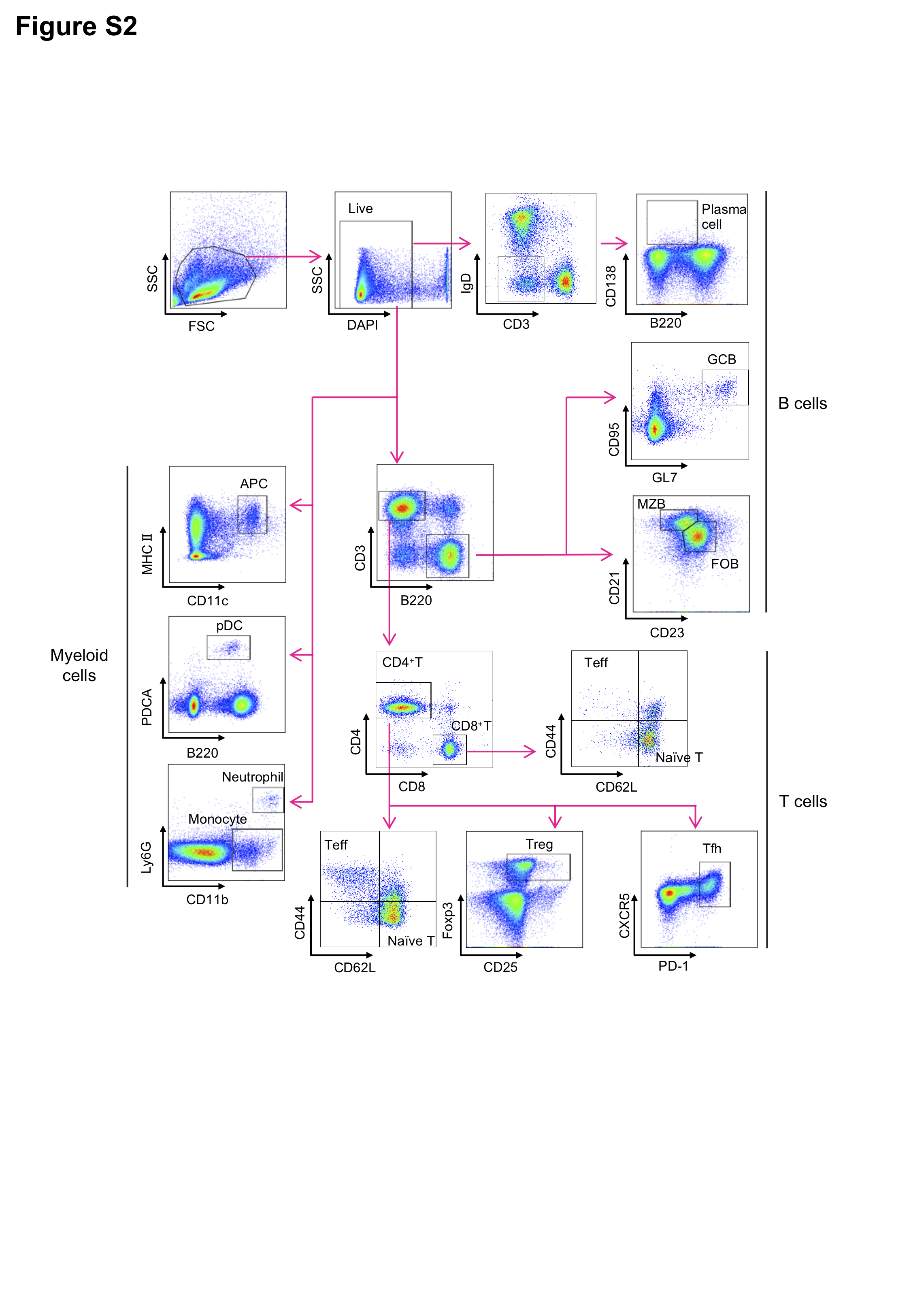

Supplement: Supplementary file 3 [file Image_2.tiff]

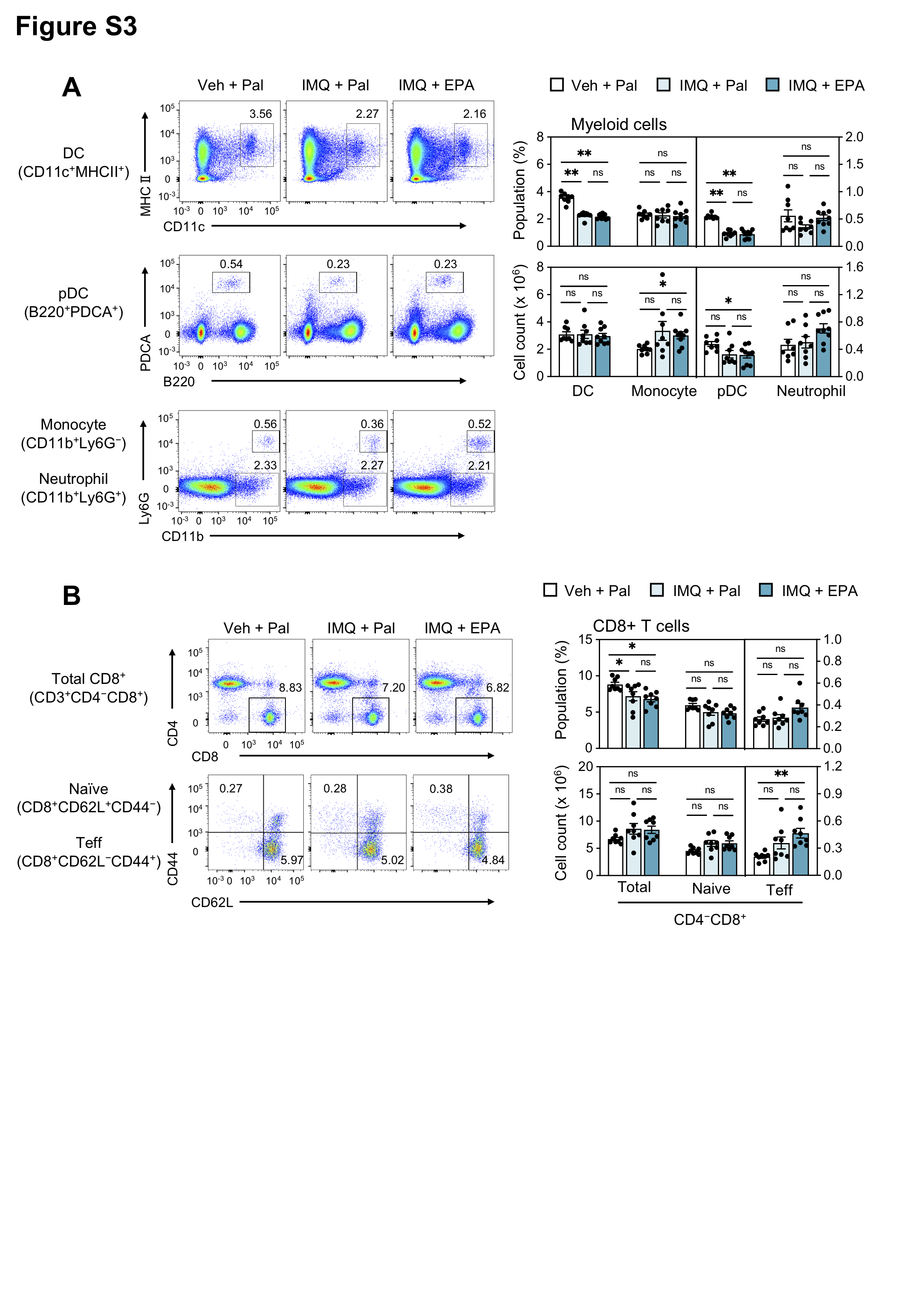

Supplement: Supplementary file 4 [file Image_3.tiff]

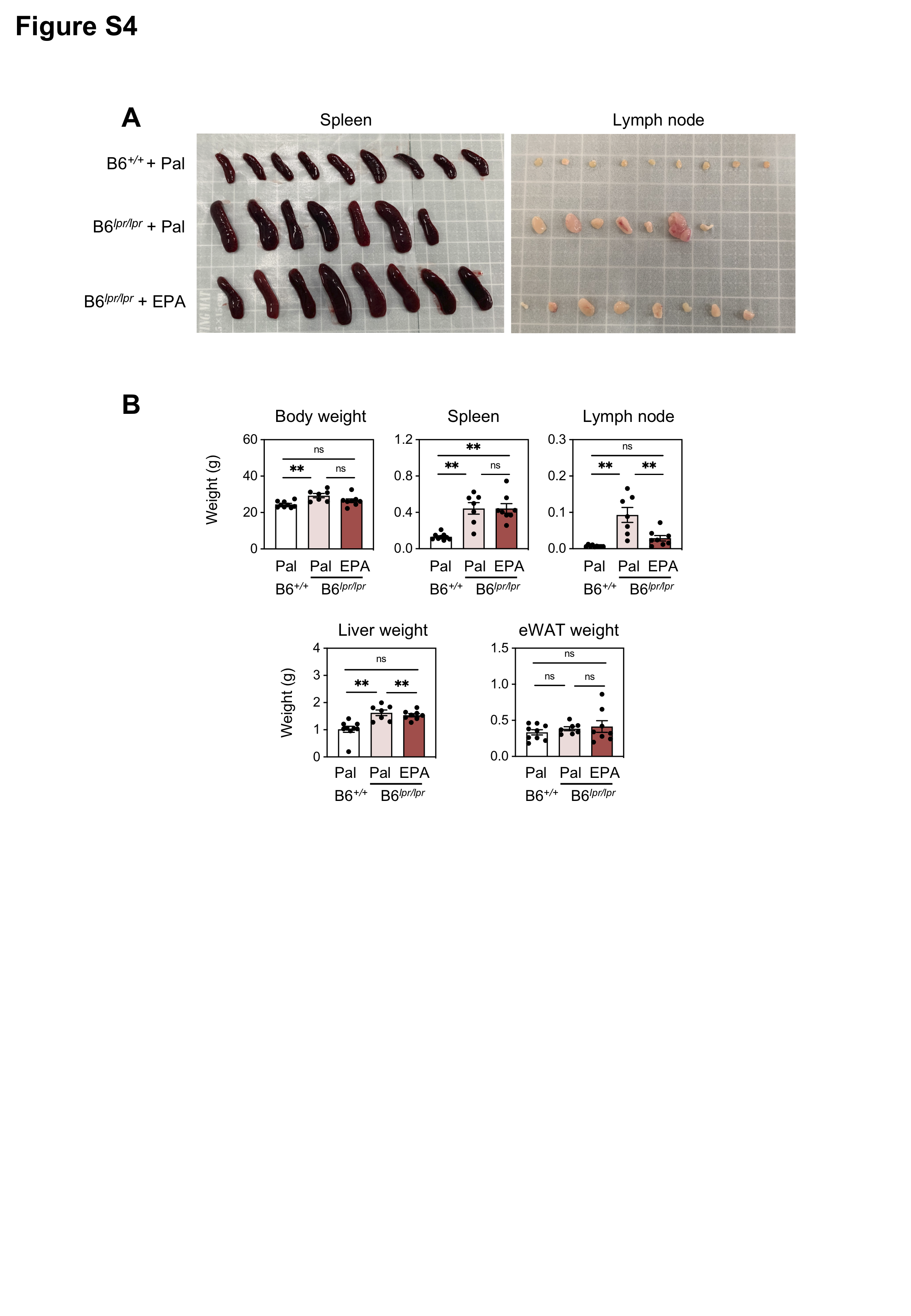

Supplement: Supplementary file 5 [file Image_4.tiff]

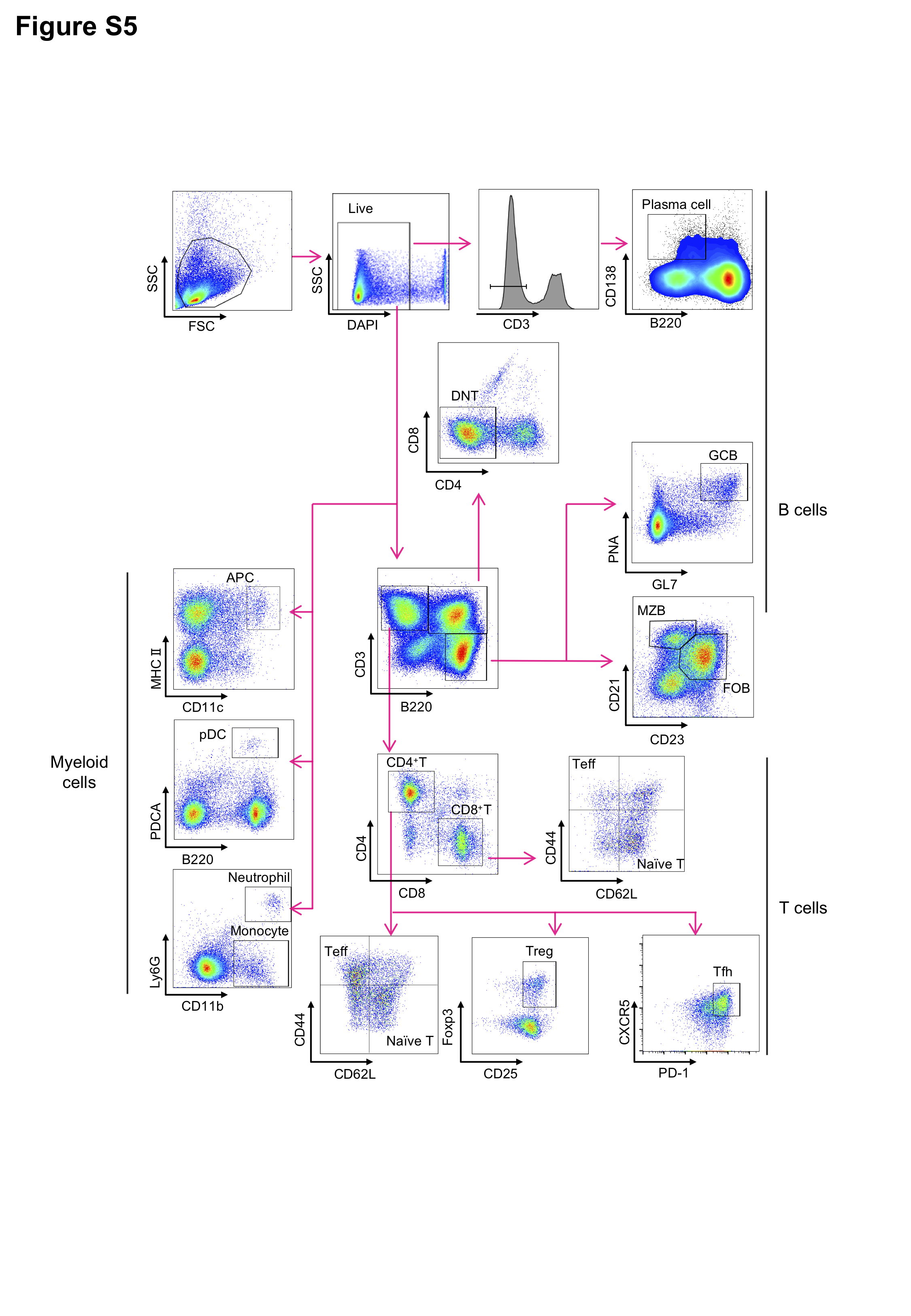

Supplement: Supplementary file 6 [file Image_5.tiff]

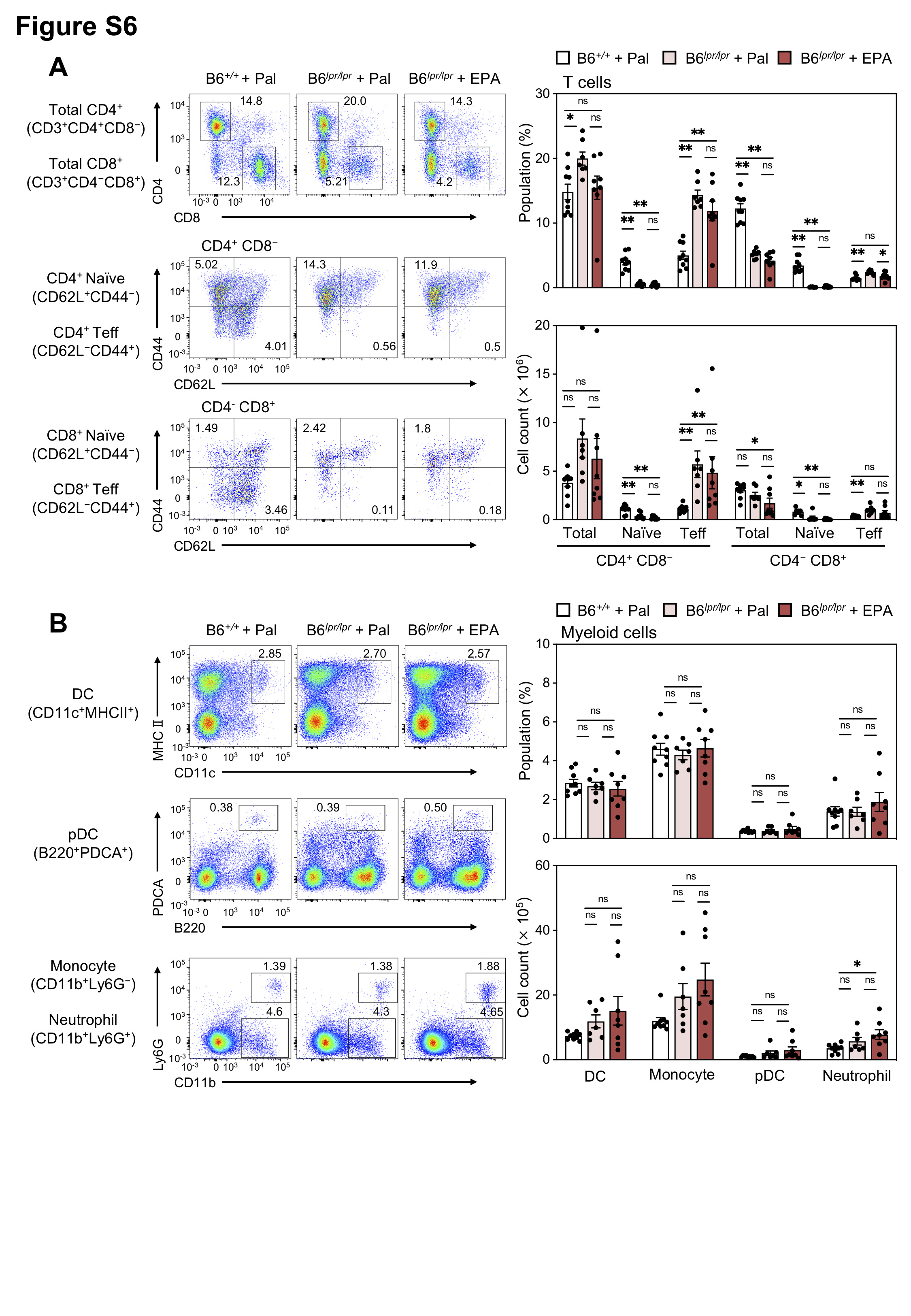

Supplement: Supplementary file 7 [file Image_6.tiff]
